# Enabling community-based metrology for wood-degrading fungi

**DOI:** 10.1186/s40694-020-00092-2

**Published:** 2020-03-19

**Authors:** Rolando Perez, Marina Luccioni, Rohinton Kamakaka, Samuel Clamons, Nathaniel Gaut, Finn Stirling, Katarzyna P. Adamala, Pamela A. Silver, Drew Endy

**Affiliations:** 1 Department of Bioengineering, Schools of Engineering and Medicine, Stanford University, Room 252, Shriram Center, 443 Via Ortega, Stanford, CA 94305 USA; 2 Department of MCD Biology, University of California, Santa Cruz, 1156 High Street, Santa Cruz, CA 95064 USA; 3 Department of Chemistry and Molecular Biophysics, California Institute of Technology, 1200 E. California Blvd, MC 138-78, Pasadena, CA 91125 USA; 4 Department of Control and Dynamical Systems, California Institute of Technology, 1200 E. California Blvd, MC 138-78, Pasadena, CA 91125 USA; 5 Department of Genetics, Cell Biology, and Development, College of Biological Sciences, University of Minnesota, 420 Washington Ave. SE, 5-178 MCB, Minneapolis, MN 55455 USA; 6 Department of Systems Biology, Harvard Medical School, 200 Longwood Avenue, Warren Alpert Building, Boston, MA 02115 USA; 7 Wyss Institute for Biologically Inspired Engineering, Harvard University, 200 Longwood Avenue, Warren Alpert Building, Boston, MA 02115 USA

**Keywords:** Synthetic biology, Mushrooms, Applied mycology, Biometrology, Literacy, Citizenship

## Abstract

**Background:**

Lignocellulosic biomass could support a greatly-expanded bioeconomy. Current strategies for using biomass typically rely on single-cell organisms and extensive ancillary equipment to produce precursors for downstream manufacturing processes. Alternative forms of bioproduction based on solid-state fermentation and wood-degrading fungi could enable more direct means of manufacture. However, basic methods for cultivating wood-degrading fungi are often ad hoc and not readily reproducible. Here, we developed standard reference strains, substrates, measurements, and methods sufficient to begin to enable reliable reuse of mycological materials and products in simple laboratory settings.

**Results:**

We show that a widely-available and globally-regularized consumer product (Pringles™) can support the growth of wood-degrading fungi, and that growth on Pringles™-broth can be correlated with growth on media made from a fully-traceable and compositionally characterized substrate (National Institute of Standards and Technology Reference Material 8492 Eastern Cottonwood Whole Biomass Feedstock). We also establish a Relative Extension Unit (REU) framework that is designed to reduce variation in quantification of radial growth measurements. So enabled, we demonstrate that five laboratories were able to compare measurements of wood-fungus performance via a simple radial extension growth rate assay, and that our REU-based approach reduced variation in reported measurements by up to ~ 75%.

**Conclusions:**

Reliable reuse of materials, measures, and methods is necessary to enable distributed bioproduction processes that can be adopted at all scales, from local to industrial. Our community-based measurement methods incentivize practitioners to coordinate the reuse of standard materials, methods, strains, and to share information supporting work with wood-degrading fungi.

## Background

The contiguous United States (U.S.) contains approximately 700 million tons of potential lignocellulosic biomass [1]. The available biomass is generally a mixture of forest and agricultural residues, urban organic waste, and algae. Bioproducts derived from lignocellulosic biomass, such as chemicals, enzymes, bioplastic bottles and packaging, and textiles contributed an estimated $393 billion and 4.2 million jobs to the U.S. economy in 2014 [2–4]. The U.S. Department of Agriculture (USDA) reports that global revenues from bio-based chemicals alone will exceed $500 billion dollars per year by 2025 [5]. Further development of infrastructure for utilizing distributed biomass resources could help enable a “Bio-Belt” that revitalizes rural manufacturing and benefits historically-disenfranchised communities [6].

Current biomanufacturing processes typically focus on microbial conversion of lignocellulosic biomass feedstocks to sugars by way of enzymatic hydrolysis followed by fermentation into higher-value products [7]. Such approaches require resource-intensive biomass preprocessing, liquid bioreactors, and ancillary equipment [2]. In contrast, wood-degrading filamentous fungi—evolved to grow directly from lignocellulose—could provide a more straightforward means of manufacture that is less resource intensive and expands what bioproducts can be made [8–11]. The direct use of wood-degrading filamentous fungi could also facilitate a more ‘circular’ economy supporting sustainable food production, manufacturing, and energy generation [12]. Direct use of wood-degrading fungi could even enable new opportunities for innovation in the global economy, especially in developing countries [12–14].

Fungi form diverse relationships with viruses, algae, plants, animals, insects, and bacteria, and are critical to human health [15–21]. Filamentous fungi, in particular, are distinguished by their multinucleate cells, known as hyphae, chitinous-composite cell walls, vast repertoire of degradative enzymes and secondary metabolites, and interconnected networks of cells, known as mycelium, that form through a combination of apical growth, hyphal branching, and regulated self-fusion [22–31]. Growing mycelium penetrate and degrade the substrate upon which the organism lives and uptake and shuttle nutrients throughout complex hyphal networks [23].

Mushrooms, the reproductive structures of wood-degrading filamentous fungi, are widely-used as foodstuffs and as a source of medicines and are consequentially revered by many cultures [32–34]. Additionally, mycological materials made from the tissues of mushrooms are used in meat replacements, filtration and remediation of contaminated water, packaging materials, furniture, textiles for the fashion industry, architectural design, art, materials for super capacitors and batteries, anti-viral therapeutics for bees, and nanoparticle synthesis [35–46]. Most commercial mycological materials based on solid-state fermentation are made by placing an organism and substrate mixture into a preformed mold and allowing the mycelium to process the substrate via solid-state fermentation. The process itself is typically halted via thermal inactivation followed by downstream processing.

Recent studies showcase the impacts that choice of organism, strain, and substrate composition have on the macroscopic properties of mycelium-based materials [47–54]. For example, Haneef et al. showed that choice of organism can change the macroscopic properties of mycelium materials, including morphological, chemical, or mechanical characteristics [52]. As a second example, Appels et al. demonstrated that genetic differences among strains of the species can also change the macroscopic properties of mycelium-based materials [50].

For broader context, Cerimi et al. recently reported that 27 different fungal species as being used in developing mycelium-based materials and processes [55]. Most organisms are of the class Agaricomycete in the division Basidiomycota. The Agaricomycete are less-extensively studied and developed as some of their Ascomycota counterparts, such as *Saccharomyces cerevisiae*, *Neurospora crassa*, or A*spergillus niger* [56–58]. The model mushrooms that have been advanced in academic research, such as *Coprinopsis cinera* for mushroom genetics, *Agaricus bisporus* for food, *Schizophyllum commune* for mushroom morphogenesis*,* and *Phanerochaete chrysosporium* for lignocellulosic biomass degradation, are different from the organisms that are used by industry, such as *Ganoderma lucidum* and *Trametes versicolor* [11, 59–67].

A wide variety of substrates supporting fungal growth are also reported throughout the academic literature [55]; substrates used by industry are often proprietary recipes that are not shared. Substrate composition has been demonstrated to be critical in defining the macroscopic properties of mycological materials. For example, Elsacker et al. demonstrated that substrate physical characteristics (e.g., fiber type) may have a greater impact on macroscopic material properties than the chemical composition of the substrate itself [68]. From their experiences Elsacker et al. declare that substrate composition and processing standards are needed.

Reliable reuse of materials and measures has long been recognized as essential for commerce and civilization [69–71]. For example, Sect. 8 of the United States (U.S.) Constitution grants Congress the power to, “fix the Standard of Weights and Measures” [72]. Standards are so useful that the U.S. Commerce Department operates the National Institute of Standards and Technology (NIST) to establish essential measures for the U.S. economy. Additionally, multiple industries support sector-specific standards-setting organizations including the International Electrotechnical Commission (IEC) for electrical and electronic engineering standards, the Internet Engineering Task Force (IETF) for information networking standards, and the International Organization for Standardization (ISO) for materials, products, processes, and services standards. Beginning in 1999, reuse of standard measures, materials, and methods began to prove useful for synthetic biology; but, the resulting impacts of standardization with and of living matter have not yet matched other technology-enabled sectors [73–80]. Although humans have long-partnered with filamentous fungi there are yet to emerge any standards supporting the coordinated reuse of wood-degrading fungi in mycologically-based bioproduction [81–84].

Ecologists studying the natural behaviors of wood-degrading fungi have long sought to standardize measurements of soil decomposition for scientific purposes. For example, ecologists have used Popsicle® sticks partially buried in soil to measure fungal growth and wood decomposition rates on a semi-homogeneous wood substrate across different ecosystem settings [85, 86]. Methods of this type have been adapted and formalized into standardized products for various applications, such as the American Society for Testing and Materials International Standard D1413-07, “Standard Test Method for Wood Preservatives by Laboratory Soil-Block Cultures” [87]. Adapted Popsicle® stick-based methods are still being used in soil studies [9]. As a second example, Keuskamp et al. developed a “Tea Bag Index” method using Lipton® tea bags to quantify soil decomposition rates and litter stabilization; bags are buried in soil and retrieved for analysis after at least four days [88, 89]. The Tea Bag index is being used to support citizen-science based measurements of plant matter decay [90, 91]. Separately, the use of consumer products in culturing wood-degrading fungi is being further explored; for example, Peeps®—a marshmallow-based food product—were used to study the survival and growth of fungi in harsh environments [92].

Here, we sought to develop materials, measures, and methods that begin to support community-based metrology for wood-degrading fungi, with an initial focus on basic laboratory culture. So-realized such methods could underlie subsequent development of additional standards supporting downstream production processes. Specifically, we sourced sequenced strains of wood-degrading fungi and used standard plate-based colony radial extension assays to determine strain performance. We show that growth performance of fungi on media made from a widely available consumer product (Pringles™) correlates well with performance on a fully characterized and traceable reference material. We also demonstrate via interlaboratory experimentation that measurements of wood-fungi performance can be coordinated across many locations.

## Results

Most mycelium-based fabrication processes use organisms whose genomes have not been sequenced. Going forward, working with sequenced organisms is absolutely critical to support science- and technology-enabled improvements in strains and manufacturing processes. Thus, in getting started, we worked to source five sequenced strains of industrially-relevant wood-degrading fungi: *Pleurotus ostreatus,* an important gourmet mushroom that is used in mycelium-materials; *S. commune,* a widely-recognized model organism for mushroom morphogenesis; *T. versicolor,* a medicinal mushroom used for remediation and mycelium materials; *G. lucidum*, a medicinal mushroom and favorite of the nascent mycelium materials industry, and *P. chrysosporium*, a recognized model organism for processing lignocellulosic biomass.

Obtaining sequenced strains for each selected organisms was complicated. For example, we first acquired a sample of *P. ostreatus* (strain ID PC-9) from the Spanish Type Culture Collection (CECT). Acquiring samples of *P. ostreatus* from CECT required completing an ad hoc materials transfer agreement, USDA Animal and Plant Health Inspection Service (APHIS) permitting, Spanish Customs export compliance requirements which included affirming future compliance with the Convention on Biological Diversity and the Nagoya Protocol, and finally U.S. Customs import requirements. However, upon first use of the so-sourced sample, we observed microbial contamination in our stock cultures, which we were able to clear through subsequent sub-culture. We experienced similar challenges acquiring sequenced strains of *G. lucidum* from various sources. We eventually acquired *G. lucidum* (10597-SS1)*, T. versicolor* (FP101664-Spp)*, P. chrysopsporium* (RP-78) from the Reference Culture Collection at the Center for Forest Mycology at the USDA Forest Service Forest Products Lab. We acquired *S. commune* 4.8B from Han Wosten. All strains except for *S. commune* 4.8B have been sequenced by the U.S. Department of Energy Joint Genome Institute (JGI). *S. commune* H4-8, a close relative of *S. commune* 4.8B, has also been sequenced. Genome assemblies can be accessed via the JGI MycoCosm portal (mycocosm.jgi.doe.gov) (see Additional file 1: Table S1) or via the National Center for Biotechnology Information (NCBI) GenBank database (*G. lucidum*: PRJNA68313, *T. versicolor*: GCA_000271585.1, *P. chrysosporium*: AADS00000000.1, *S. commune*: GCA_00014385.1).

Importantly, all organisms as acquired are not on the U.S. Regulated Plant Pest list, which makes reuse simpler for U.S.-based practitioners. International regulatory frameworks governing biomaterial transport may present administrative and logistical challenges for coordinating work with wood-degrading fungi, especially for low-resourced practitioners, and may hinder broader adoption of standard materials, measures, and methods. One possible scenario we envision that could support coordination across locations is for well-resourced practitioners to bank international reference strains and correlate their performance on reference substrates with the performance of locally-sourced strains on the same reference substrates. Such practices could enable the performance of locally-sourced strains to be compared with international reference strains.

We then worked to confirm a suitable measurement method that can be readily used by a diversity of practitioners. First, we compared total biomass accumulation in liquid culture with plate-based measurements of colony radial extension to determine if either method would be practical for community-based measurements. Liquid-culture methods do not resemble the ambient conditions encountered in solid-state fermentation, typically take more than 5 days to complete, use sample volumes greater than 10 mL, and require ancillary equipment to properly culture the samples, harvest and dry the biomass, and measure the dry cell weight. In our small pilot studies, we found 5 mL culture volumes of various fungi and substrate combinations resulted in irreproducible results, and so we abandoned a liquid culture-based measurement method.

**Fig. 1:**
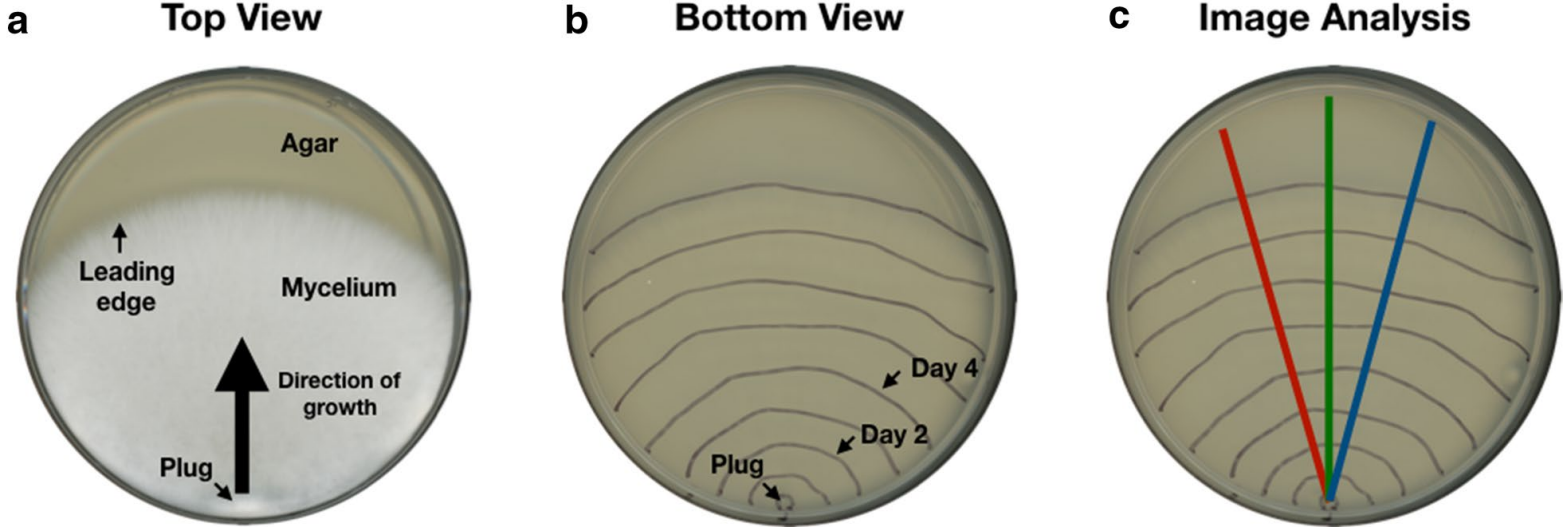
Plate-based radial extension measurements are an accessible method for quantifying the performance of wood-degrading fungi. **a** The fungal colony is started from a single inoculation point of fungal tissue. The 5 mm tissue plug is taken from a common source plate of fungal tissue, applied to an experimental plate, and incubated in darkness at 30 °C; resulting in leading edge radial growth along the agar surface in the direction indicated by the large black arrow. **b** The leading-edge of the growing colony is traced every 24 h with a marker. **c** On the final day traces are imaged and analyzed using ImageJ. Measurements are made along each axis, red, green, and blue at intersections with the leading edge traces. A linear regression model is computed for each sample (plate) using all data for all three axes for that sample. The coefficient of the model defines the radial extension rate for that sample

Plate-based colony radial extension measurement methods more closely reflect the ambient conditions of solid-state fermentation processes and require less equipment than liquid-based methods [48]. While plate-based methods do not directly quantify total fungal biomass, such methods can serve as an important first-step toward developing standard measures and methods that might later be expanded to more closely reflect solid-state fermentation conditions. Furthermore, a simple and accessible method such as a plate-based assay can incentivize the early adoption of standard materials, measures, and methods for working with wood-degrading fungi across a variety of resource settings. The plate-based colony radial extension rate measurement method consists of daily traces of the colony radius, followed by imaging of the traces, analysis of the images to quantify daily radial growth along each of three equally-spaced axes, followed by linear regression modeling of all data for individual samples to calculate extension rates (Fig. 1). In addition to quantifying colony radial extension rates, we observed qualitative differences in hypha density and hyphal branch rate between various fungi and substrate combinations (see Additional file 1: Figure S1).

**Fig. 2:**
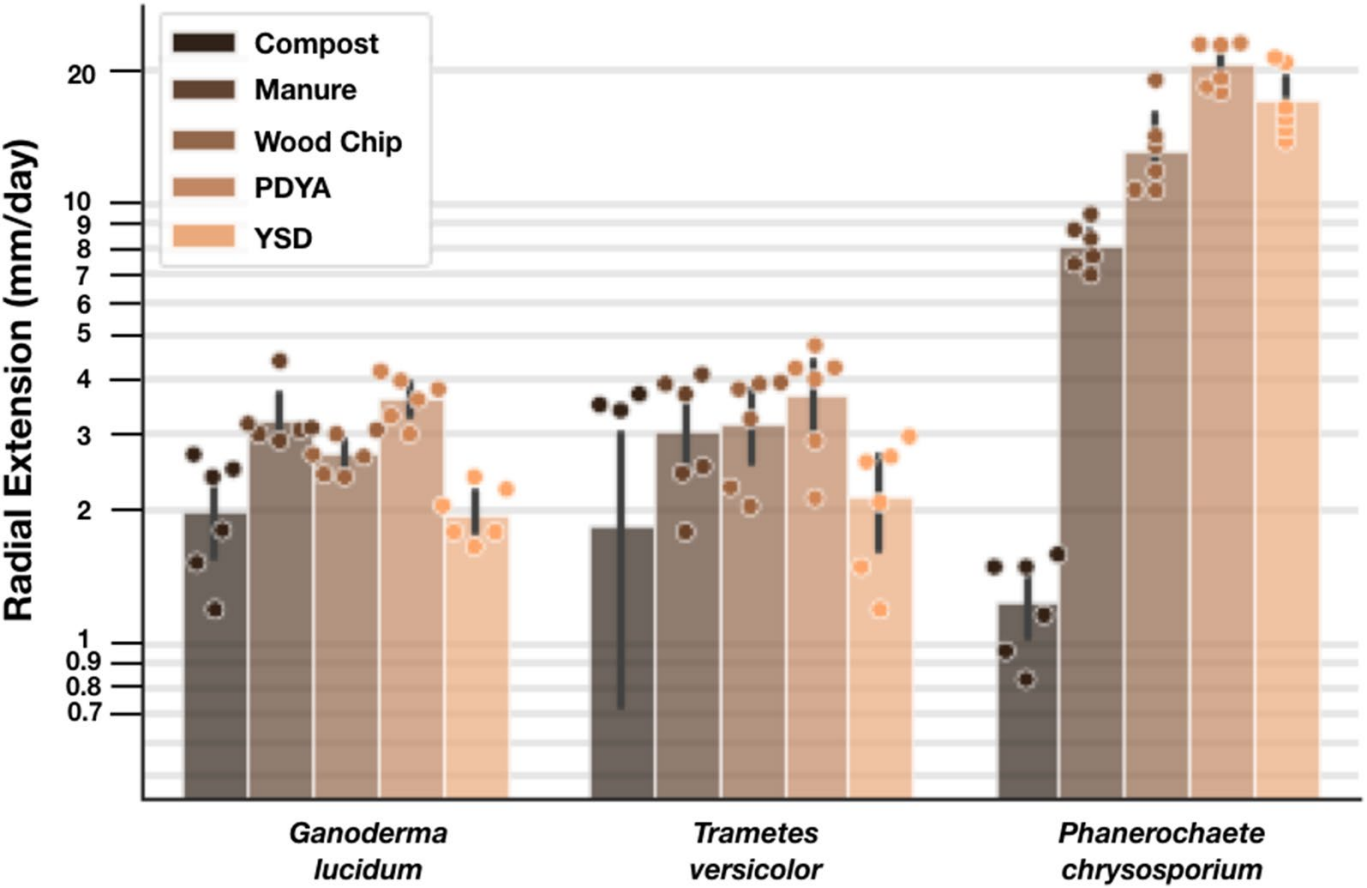
Extension rates of selected wood-degrading fungi vary up to 7.5-fold with respect to substrate composition. We measured radial extension rates of three different sequenced wood-degrading fungi on five different substrates. *P. chrysosporium* had the greatest average extension rate followed by *T. versicolor,* and *G.* lucidum. Rates below 2 mm/day are shown but are considered to be at the limit of our measurement technique. Statistically significant differences were detected by way of Tukey’s HSD (p < 0.05) for *P. chrysosporium* and *T. versicolor*, and *P. chrysosporium* and *G. lucidum.* Statistically significant differences were detected by way of Tukey’s HSD (p < 0.05) for Compost and Wood Chip, Manure PDYA, and YSD; between Manure and PDYA; and between PDYA and Wood Chip. Radial extension rates for individual plates (points, n = 6 for all conditions except for *T. versicolor* on compost, n = 3), mean extension rates across all plates (bar height), and standard deviation across all plates (error bar) are shown

Wood-fungus are often grown on “found” feedstocks that can be sourced locally. To better understand the range of growth performance as a function of variation in feedstocks we measured fungal colony growth rates for *G. lucidum, T. versicolor,* and *P. chrysosporium* on agar-based substrates made from aqueous extracts of five locally-available substrates: plant compost from the Stanford Farm, horse manure from the Stanford Barn, a commercially-available wood chip substrate, laboratory-grade potato dextrose yeast extract (PDYA), and a yeast synthetic defined media (YSD) (see Additional file 1: Table S2). We found that all fungi tested grew on all substrates (Fig. 2). However, extension rates varied up to threefold across organisms and up to 7.5-fold across substrates. *P. chrysosporium* had the greatest average extension rate followed by *T. versicolor,* and *G.lucidum*.

Differences in growth among strains and across feedstocks make it difficult to compare measures of performance across locations and over time. One impractical approach to enabling comparable measurements would be to regularize all feedstocks and strains. Instead we sought to identify a reference material that could be used as a standard substrate for calibrating wood fungus growth. Practitioners might make measurements of growth on both the standard and local substrates, allowing for comparison of performance in relation to a standard substrate used in common. For example, Eastern Cottonwood Whole Biomass Feedstock (NIST RM 8492) was developed as a standard lignocellulosic reference material supporting biomass research. RM 8492 has undergone full compositional characterization, has been used in NIST-led interlaboratory studies, and is available for purchase as a calibration tool for biomass feedstock production facilities (see Additional file 1: Table S3) [93, 94]. However, the material is only available in limited quantities and expensive ($8.26/g).

We thus sought standard substrates that are low-cost, can be sourced by anyone anywhere, and, if possible, related to NIST RM 8492. To do so we sourced materials that might support wood-fungus growth and that can be found throughout global consumer-product supply chains. Specifically, we sourced cardboard from Amazon Prime™ shipping boxes, wood chips from hard- and soft- wood shipping pallets, and Pringles™—a consumer food product—as candidate standard substrate materials. To elaborate on the motivations guiding our sourcing process via a detailed example: Pringles™ are a standardized potato-based food that costs ~ $0.01/gram and is available in approximately 140 countries [95] (whereas Peeps® and Lipton® tea bags are only available in two and 110 countries, respectively); Pringles™ generated a reported $342 Mio. in retail sales in the UK alone in 2018, a scale at which economic incentives ensure that Pringles™ remain available regardless of global and regional politics [96]; Pringles™ themselves are composed primarily of potato starch and are produced to specification by only five factories globally (Malaysia, Belgium, China, Poland, U.S.); Pringles™ shipments typically arrive on standardized pallets and within standardized cardboard tubes.

**Fig. 3:**
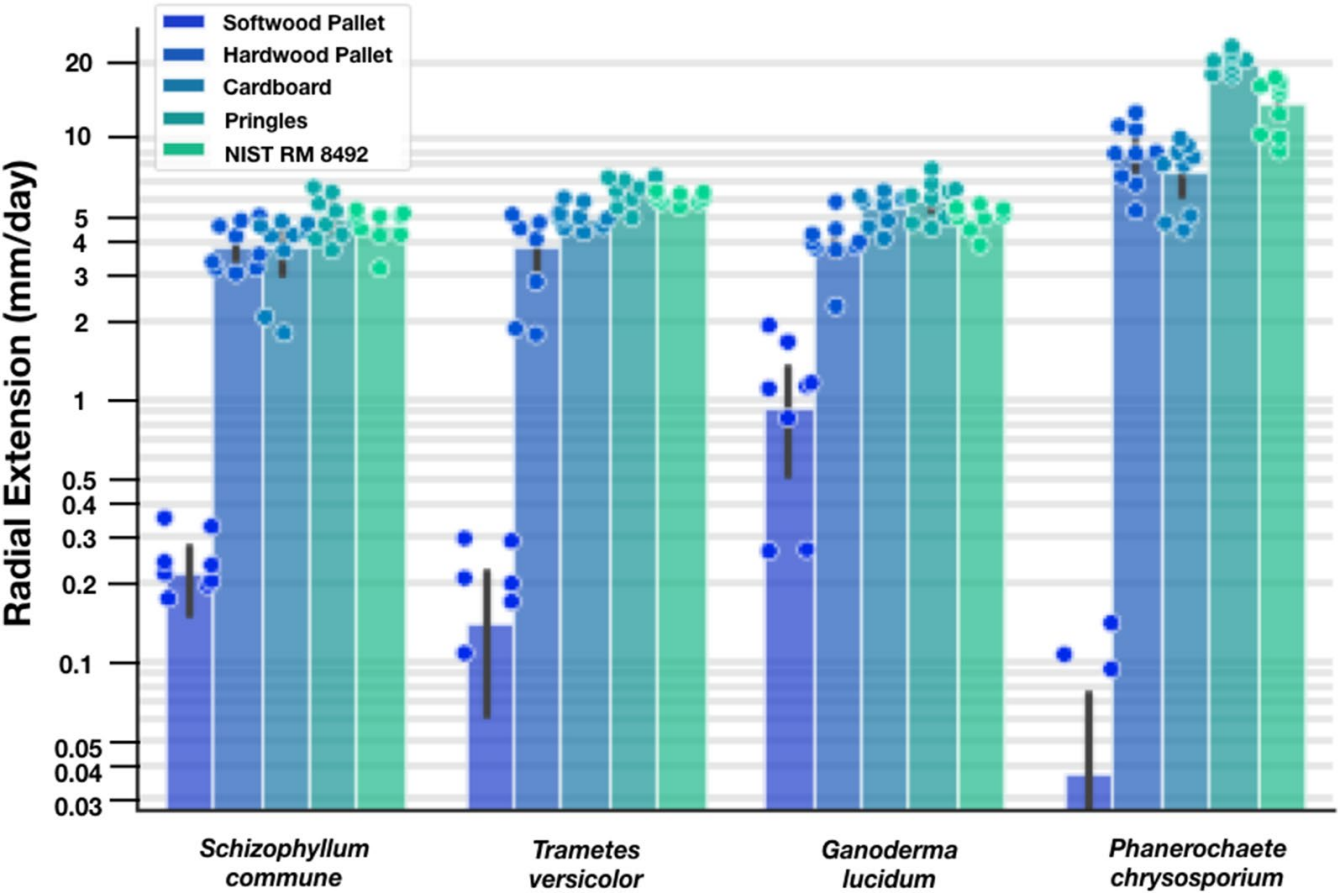
Pringles™ are a low-cost readily-available material supporting growth measurements of wood-degrading fungi. We measured radial extension rates of four select sequenced strains of industrially relevant wood-degrading fungi on five materials that can be found at the edges of global shipping networks. Extension rates vary up to 4-fold across various consumer materials.* P. chrysosporium* had the greatest average extension rate, followed by *G. lucidum*, *T. versicolor*, and *S. commune.* Rates below 2 mm/day are shown but are considered to be at the limit of our measurement technique. Statistically significant differences were detected by way of Tukey’s HSD (p < 0.05) for *P. chrysosporium* and all other strains. Statistically significant differences were detected by way of Tukey’s HSD (p < 0.05) for Cardboard and Softwood Pallet, Pringles™, and NIST RM 8492; between Hardwood Pallet and Softwood Pallet, Pringles™, and NIST RM 8492; between Pringles™ and NIST RM 8492, and Softwood Pallet; and NIST RM 8492 and Softwood Pallet. Radial extension rates for individual plates (points, n = 6 for all conditions except for *P. chrysosporium* on Softwood Pallet, n = 3), mean extension rates across all plates (bar height), and standard deviation across all plates (error bar) are shown

We tested the performance of four industrially-relevant wood-degrading fungi—*S. commune*, *T. versicolor*, *G. lucidum*, and *P. chrysosporium—*on the candidate widely-available substrates. We observed that Pringles™ supported the greatest extension rate across all organisms tested (Fig. 3). We also determined that extension rates varied up to 2.5-fold across the different organism and substrate combinations. *P. chrysosporium* had the greatest average extension rate, followed by *G. lucidum*, *T. versicolor*, and *S. commune.* For the four fungi tested, aqueous extracts of woodchips made from softwood pallets exhibited the slowest extension rates; rates below 2 mm per day are shown but are below the precision limit of our measurement technique.

**Fig. 4:**
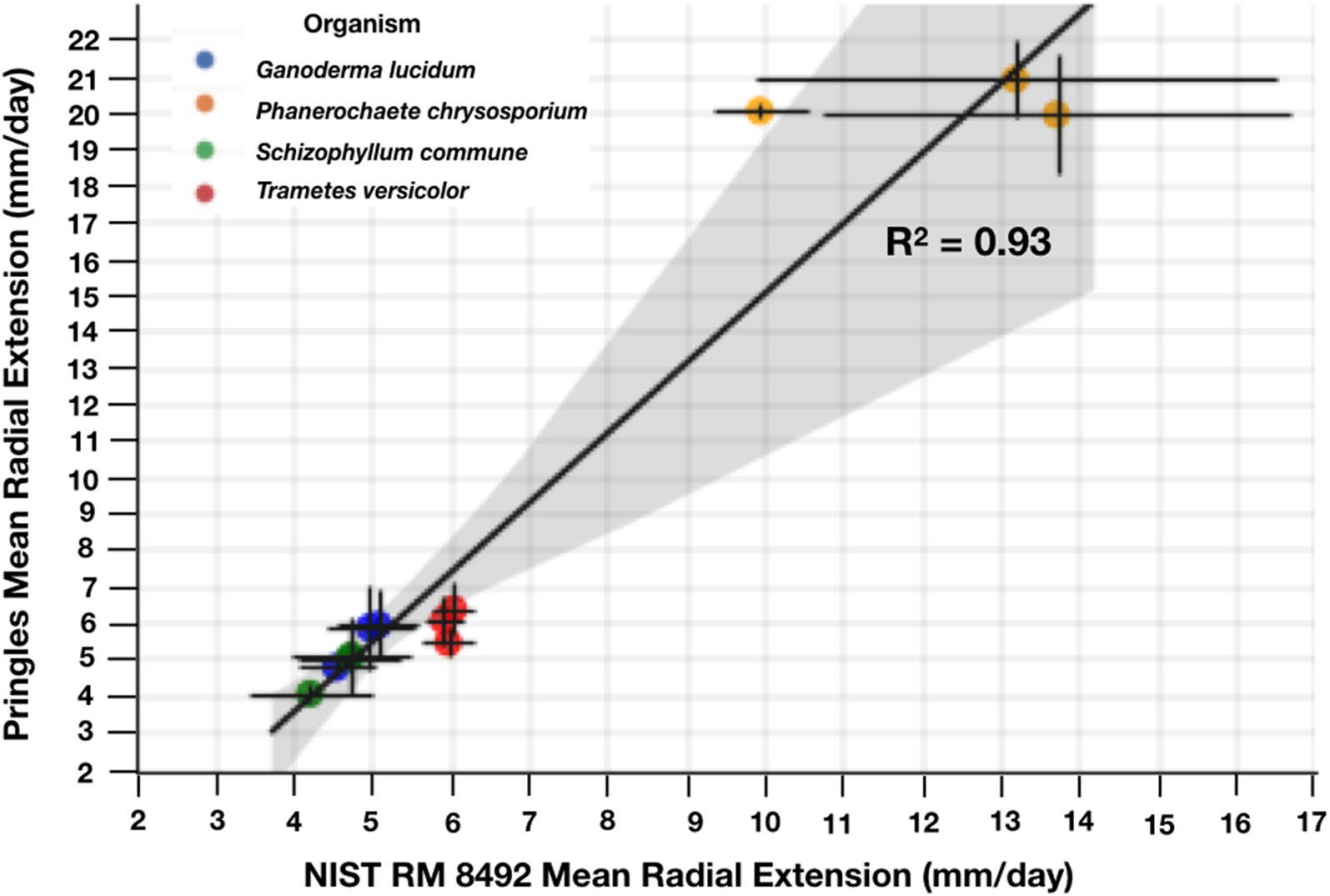
Pringles™ and NIST RM 8492 extension rates are well correlated (R^2^ = 0.93, n = 3). The x- and y- components of each data point indicate the mean radial extension rate in millimeters per day for a given organism grown on NIST RM 8492 versus the mean radial extension rate of the organism grown on Pringles™, respectively. Replicates were grouped by substrate batch number. Error bars indicate the standard deviation in the x- and y- axis. The black line indicates the linear regression model with corresponding 95% confidence interval (shaded black)

We chose to proceed with Pringles™ as a widely-available substrate reference material primarily because it supported the most robust growth among all of the fungi tested, implying that Pringles™-based media may support the growth of a wider range of fungi. Though robust growth is not necessarily a prerequisite for substrate reference materials, the ability to support the growth of a diversity of fungi other than wood-degrading fungi could be more generally useful; Pringles™ are composed primarily of sugar-rich substances: dried potatoes, cornstarch, rice flour, and wheat starch, all of which are commonly-used substrates for mycological production. We then compared the performance of all organisms on media made from Pringles™ to performance on media made from NIST RM 8492 using a simple linear model (Fig. 4). The observed correlation (R^2^ = 0.93) supports the potential to relate measurements of wood-fungus growth made by practitioners at the edges of consumer supply-chains to measurements made by well-resourced practitioners with access to fully-traceable and compositionally-characterized materials.

**Fig. 5:**
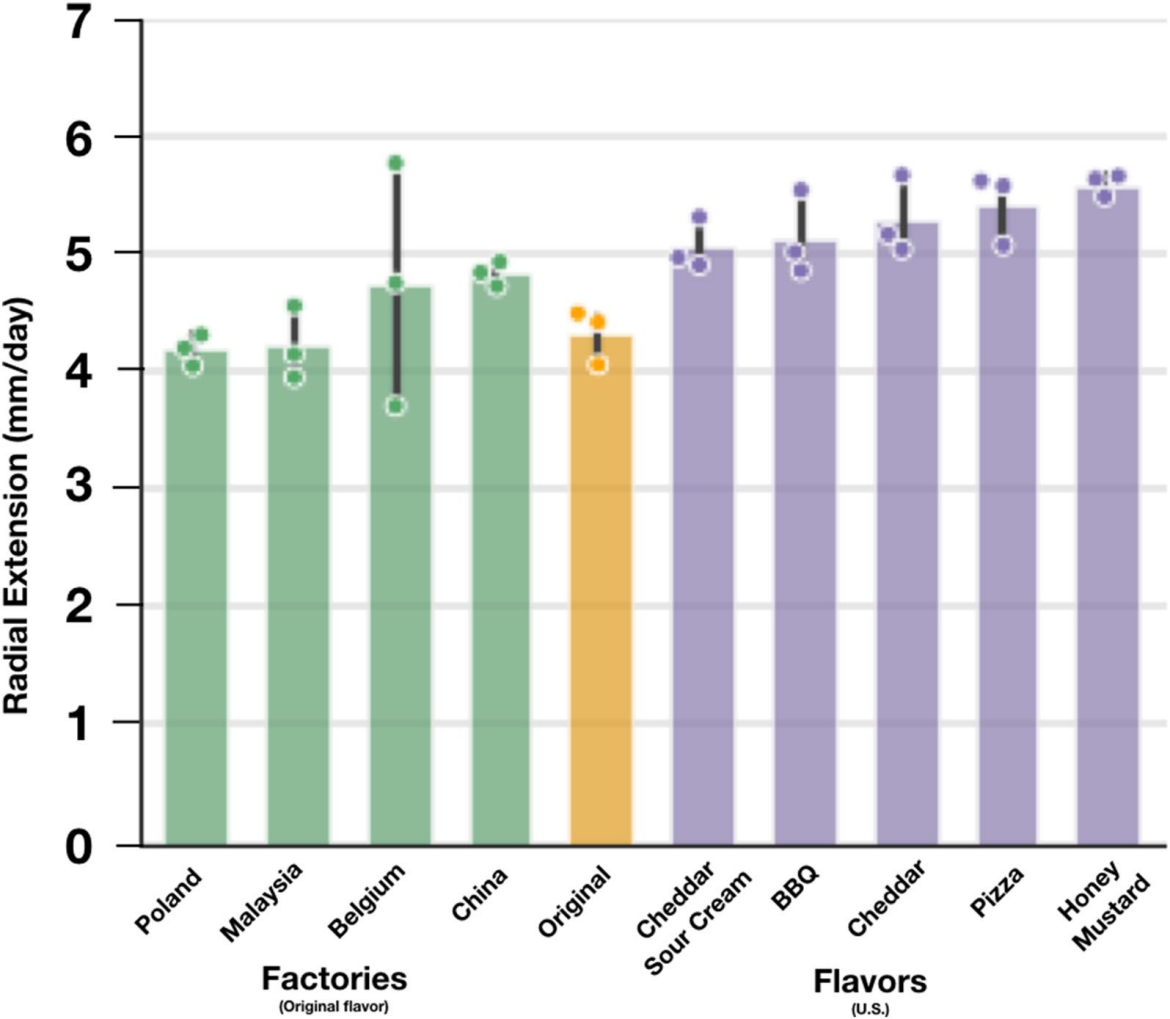
Extension rates on Original-flavor Pringles™ sourced from all five global factories and on assorted flavors from the U.S. vary less than 1.3-fold. We measured the extension rate of *Ganoderma lucidum* on substrates made from Original-flavor Pringles™ sourced from each of the five Pringles™ production factories as well as substrates made from BBQ-, Sour Cream-, Cheddar-, Honey Mustard-, and Pizza-flavored Pringles™. Original-flavor Pringles™ made in China supports the highest extension rate while Honey Mustard flavor supports the highest extension rate among the US-made Pringles™ flavors tested. No statistically significant differences were detected between production factories, flavors, or between factories and flavors (Tukey’s HSD, p < 0.05) potentially supporting their utility as standard reference materials. Radial extension rates for individual plates (points), mean extension rates across all plates (bar height), and standard deviation across all plates (error bar) are shown. n = 3 for all rates except for Original-USA, n = 4. Replicates for two groups, Original (made in TN, USA) and `USA (Original flavor, made in TN, USA), were combined into Original-USA and two plates per substrate were dropped due to microbial contamination

To further explore variation in wood-fungus growth measurements as a function of Pringles™ sourcing and composition, we measured and compared the extension rates of *G. lucidum* on substrates made from Original-flavor Pringles™ sourced directly from China, Malaysia, Poland, Belgium, and the U.S., and also from substrates made from BBQ-, Sour Cream-, Cheddar-, Honey Mustard-, and Pizza-flavored Pringles™ sourced from the U.S. (Fig. 5). Radial extension rates of *G. lucidum* on substrates sourced from the five Pringles™ production factories (Original flavor) and other Pringles™ flavors (assorted) varied ~ 1.3-fold. Original-flavor Pringles™ made in China supported the highest extension rate while Honey Mustard-flavor supported the highest extension rate among the U.S.-made Pringle™ flavors tested; observed coefficients of variation were 0.12 and 0.04 for factories and flavors, respectively.

**Fig. 6:**
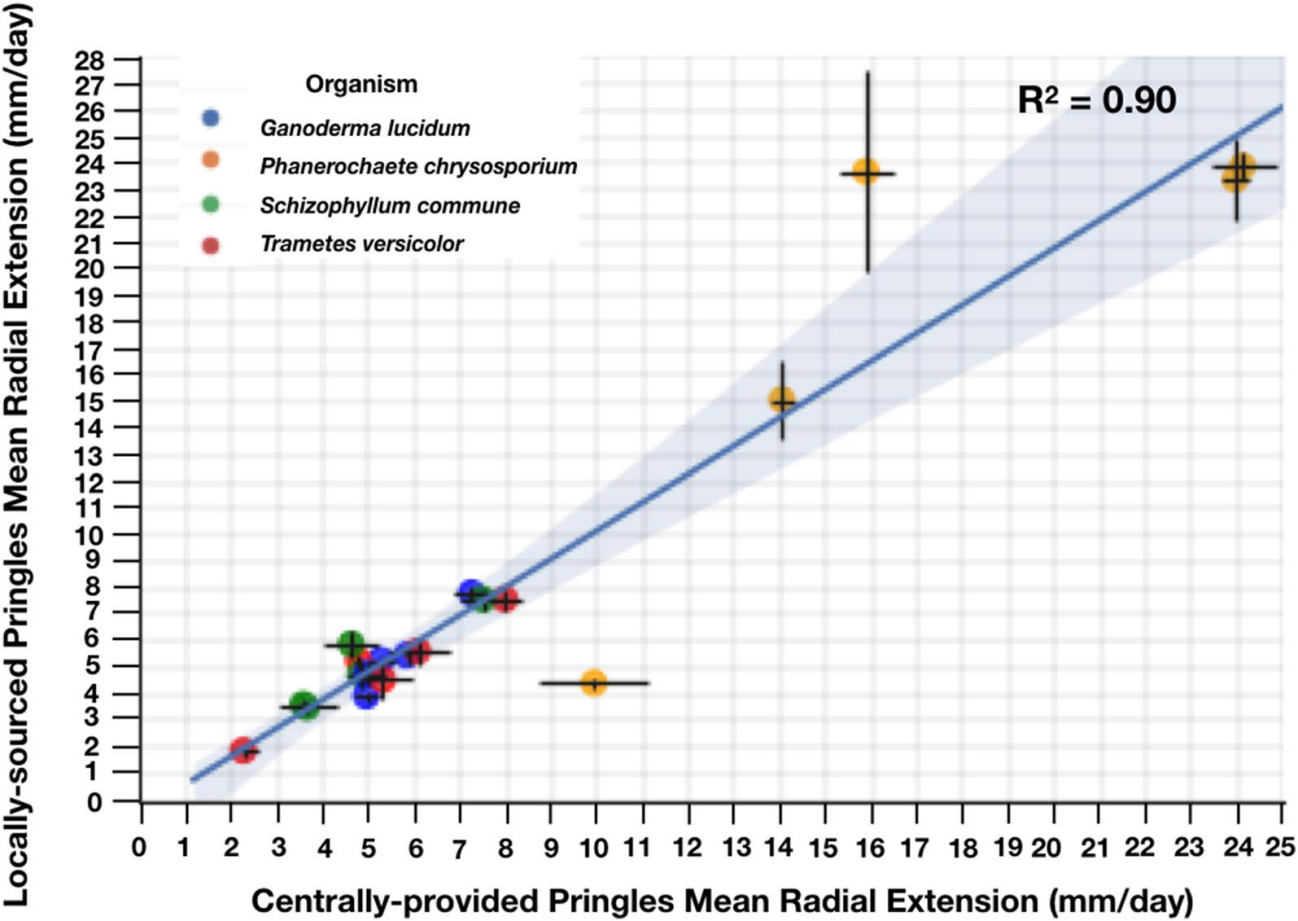
Community-based measurements of radial extension on media made from locally-sourced and centrally-provided Pringles™ are well correlated (R^2^ = 0.90, n = 3). We distributed a measurement kit containing our proposed reference materials: *G. lucidum* (10597-SS1), *T. versicolor* (FP101664-Spp)*, S. commune* (4.8B)*, P. chrysosporium* (RP-78)*,* and centrally-provided Original-flavor Pringles™. Participating labs also acquired their own local form of Original-flavor Pringles™ and used both substrate sources for radial extension experiments. Each data point represents the reported rates by each participating laboratory. The x- and y- components of each data point indicate the mean radial extension rate in millimeters per day for a given organism grown on centrally-provided Pringles™ versus the mean radial extension rate of the organism grown on locally-sourced Pringles™, respectively. Replicates were grouped by lab, organism, and substrate. Error bars indicate the standard deviation in the x- and y- axis. The blue line indicates the linear regression model with corresponding 95% confidence interval (shaded blue)

To practically explore the potential for reuse of consumer-sourced materials via a common measurement method we sent metrology kits to four participating laboratories (see Additional file 1: Figure S2). The kit included the four fungi strains of interest, a coring tool for excising fungal tissue plugs, cheese cloth for filtering aqueous extracts, centrally-provided Pringles™, and simple instructions for conducting colony radial extension measurements. Interlaboratory study participants independently acquired locally-sourced Pringles™, conducted experiments, and returned images of experimental plates for image processing and analysis. Measurements of growth rates on media made from locally-sourced versus centrally-provided Pringles™ substrates are well correlated (R^2^ = 0.90), indicating locally-sourced Pringles™ perform comparably to centrally-provided Pringles™ (Fig. 6), and were within 1.5-fold, with the exception of Lab 4 measurements of *P. chrysosporium* growth rates (see Additional file 1: Figure S3). Observed coefficients of variation for growth rates obtained in our lab were 0.11, 0.04. 0.02, 0.16 for *S. commune*, *T. versicolor*, *G. lucidum*, and *P. chrysosporium*, respectively; coefficients of variation in growth rates across all other labs were 0.23, 0.28, 0.06, and 0.39 for *S. commune*, *T. versicolor*, *G. lucidum*, and *P. chrysosporium*, respectively.

**Fig. 7:**
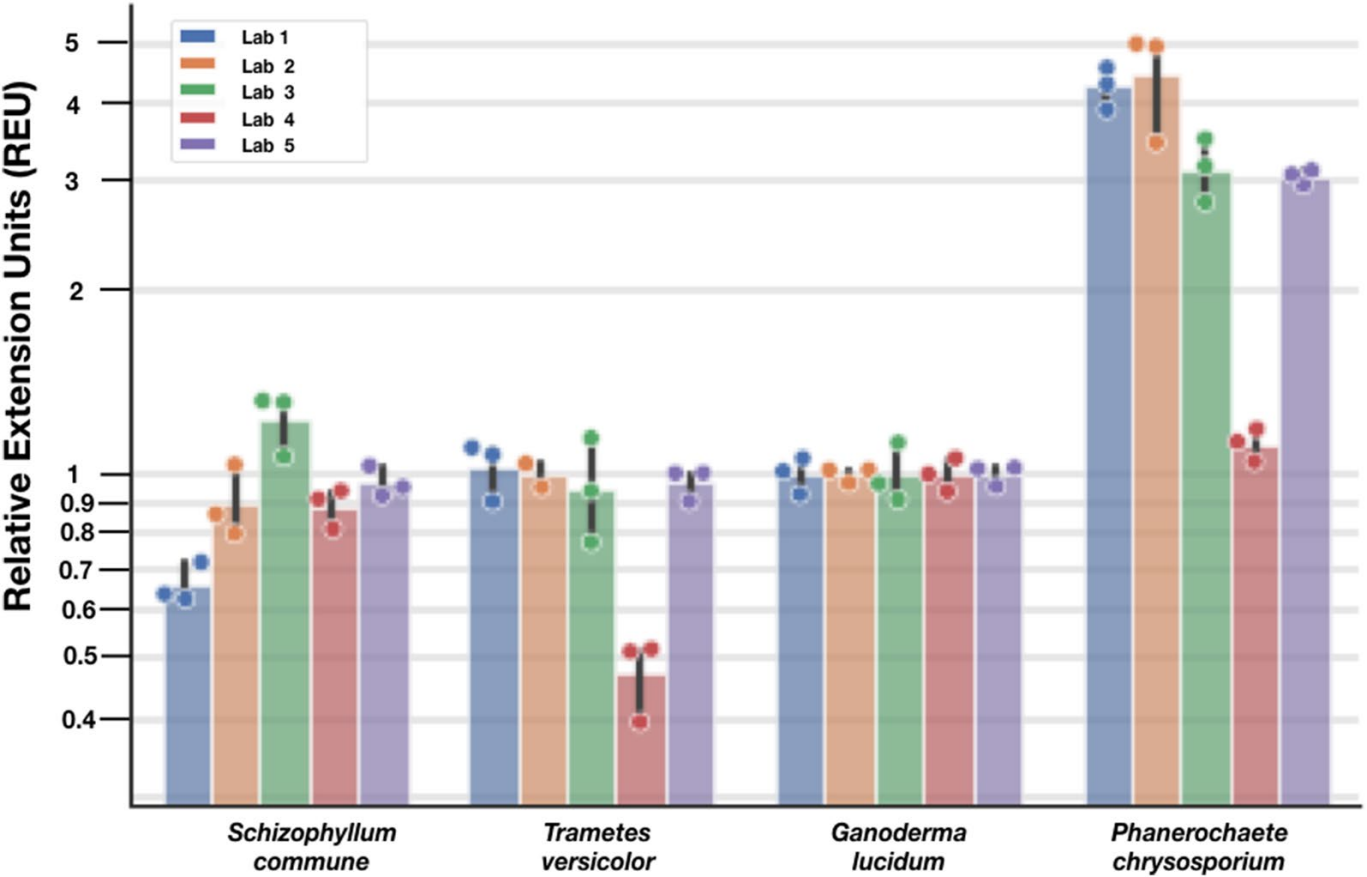
Reference materials and relative reference units improve community-based measurements of performance for wood-degrading fungi. Using the same data from Fig. 6, we divided each lab’s reported extension rates for a particular organism by the lab’s reported extension rate for *G. lucidum* grown on media made from locally-sourced Pringles™. The resulting Relative Extension Units (REU) for each organism are within 2-fold with the exception of REU reported by Lab 4 for *T. versicolor* and *P. chrysosporium*. Reporting REUs reduced coefficients of variation for *G. lucidum* by 75%, for *S. commune* by 33%, for *T. versicolor* by 35%, and for *P. chrysosporium* by 11%. Radial extension rates for individual plates (points, n = 3), mean extension rates across all plates (bar height), and standard deviation across all plates (error bar) are shown

To explore reducing variation in measurements across participating laboratories we defined relative extension units (REU) and calculated REU for all colony growth rates. In doing so we sought to leverage the use of relative units to normalize the performance of biological systems in relation to extrinsic factors that are difficult to identify or impossible to control, as has been previously established for reporting levels of gene expression [79]. Specifically, using growth rate data obtained with locally-sourced Pringles™ (Fig. 6), we divided each lab’s reported extension rates for a particular organism by the lab’s reported extension rate for *G. lucidum* on locally-sourced Pringles™ (Fig. 7). The resulting REU for each organism are within 2-fold of each other with the exception of REU reported by Lab 4 for *T. versicolor* and *P. chrysosporium.* REU for each organism using the centrally-provided Pringles™ are also within 2-fold, again with the exception of REU reported by Lab 4 for *T.* versicolor (see Additional file 1: Figure S4). Reporting via REUs reduced coefficients of variation in all cases: by 75% for *G. lucidum*, by 33% for *S. commune*, by 35% for *T. versicolor*, and by 11% for *P. chrysosporium*. Reporting REUs for experiments using centrally-provided Pringles™ reduced coefficients of variation for *G. lucidum* by 63%, for *S. commune* by 33%, for *T. versicolor* by 22%, and for *P. chrysosporium* by 32% (see Additional file 1: Figure S4).

## Discussion

We established preliminary methods and materials that enable community-based metrology for wood-degrading fungi. We did so by sourcing sequenced organisms, identifying widely-available substrate feedstocks, and correlating growth performance to traceable reference materials via a simple measurement method. Taken together, these methods and materials should begin to improve the reproducibility and reliability of fabrication processes involving mycelium materials by instantiating a culture of coordination in the wood-degrading fungi and mycelium materials research communities; and by incentivizing practitioners to adopt standard reference materials, measures, and methods.

Specifically, we demonstrated that widely-available consumer materials, such as Pringles™, can serve as an operational standard for mycological growth processes. As noted, Pringles™ can be found in most countries and are composed primarily of sugar-rich ingredients that are commonly used for mycological production. Furthermore, Pringles™ are ~ 800-fold more affordable than fully characterized and traceable reference materials (NIST RM 8492 cost $8.26/gram; Pringles™ cost ~ $0.01/g). To be clear, Pringles™ themselves would likely not be a favored source of substrate for large-scale manufacturing processes; rather, Pringles™ can serve as an affordable and widely-available reference material that can be accessed and used by many practitioners across many locations. Of course, given that Pringles™ are not developed as a fixed metrological reference material, care should be taken to periodically assess how the composition or properties of Pringles™ may change in response to corporate, consumer, or market forces. Though NIST RM 8492 can be useful for calibrating lignocellulosic analysis equipment used to characterize solid-state feedstocks, this official reference material is less accessible and prohibitively expensive for many would-be practitioners. Thus, Pringles can serve as a cheaper, more widely-available reference material that incentives the adoption of standard materials, measures, and methods for working with wood-degrading fungi and fabricating mycelium materials.

We observed that flavored Pringles™ generally supported slightly faster growth than Original-flavor Pringles™ (Fig. 5). Although we were not able to conduct compositional analysis of the flavored Pringles™ substrates, each product comes with a nutritional label and ingredients list. Many of the ingredients are the same across Pringles™ flavors, with Original flavor serving as the baseline: dried potatoes, vegetable oil, determinate yellow corn flour, cornstarch, rice flour, maltodextrin, mono- and diglycerides, salt; as are the nutritional profiles. The various flavors have different additives including: lactic acid, yeast extract, cream, citric acid, blue cheese, butter milk, turmeric extract color, onion powder, and Red 40 Lake. It is possible that one or more flavor additives may affect wood-fungus extension rates, and future work could determine any specific impacts of flavor additives on growth rate and reference substrate performance.

From an equipment perspective we believe that slightly-more sophisticated measurement tools may be useful. For example, simple flat-bed document scanners could be used to achieve time-lapse imaging of radial extension to enable more precise quantification of extension dynamics [97]. Additionally, flat-bed scanners have been used to quantify the total number of hyphal tips, total number of hyphal segments, total number of network nodes, total length of the mycelium, growth angle, and branching angle [97]. Furthermore, using time-lapse imaging could speed up determination of extension rates and reduce the size of the plates being used; transitioning from 9 cm plates to 24-well culture plates could enable increased throughput and sample numbers, and better support statistical analysis of differences in strain performance.

Improved protocols that reduce sources of variation which inevitably arise in the making of measurements across many locations are worth exploring. More specifically, the observed variation among labs may be due to differences in the handling of the organisms, the substrate, the cultivation environment, or deviations from the simple experimental protocol supplied. While we advocated for participants to start growth assays immediately upon receipt of our kit, the protocol itself did not prescribe immediate experimentation; although we sent all labs starter cultures from the same stock plate at the same time, some labs were unable to start work immediately and had to store their cultures at 4 °C until they were able to conduct the experiment, at which time they would have followed the portion of our simple protocol that explained how to prepare the experimental plates. One lab requested a second kit after their plates dried out because of prolonged storage; we sent a new kit with samples from our mother plate and provided no further instructions. Fungal colonies age with both time and sub-culturing, and such aging may have an effect on colony radial extension rates. Therefore, prolonged storage at 4 °C, delays in sampling from starter plates to make experimental plates may have an effect on radial extension rates and may contribute to the observed variation across labs. It is also possible that the observed variation is due to contamination of the stock, starter, or experimental plates; two labs reported contamination of their kits. One lab was able to clear the contamination and proceed; we observed little variation between their results and other labs. We sent a new kit to the second lab that reported contamination and they proceeded with experiments, reporting the most consistent results.

Other potential sources of variation include variation in substrate preparation, timing of colony radius traces, or effects of mutations resulting from sub-culturing. Future work could recapitulate our interlaboratory study with improved measurement methods—perhaps, using flat-bed scanners—and re-sequence the strains as part of the protocol to help elucidate the effects of mutations on radial extension rate measurements across locations. Furthermore, such work coupled with synthetic biology tools could extend the utility of using a reference strain as an internal benchmark and the REU framework to normalize away extrinsic factors affecting radial extension measurements [98]. Reuse of materials, measures, and methods should itself help with broader adoption of improved metrology practices involving filamentous fungi and help to mitigate some of the challenges ahead for mycological production.

## Conclusions

Our community-based measurement methods enable practitioners to coordinate the reuse of standard materials, methods, strains, and to share information supporting work with wood-degrading fungi. We hope our approach will help support reproducible science and the development of more reliable fabrication processes. In particular, widely-available reusable materials, measures, and methods that enable practitioners at the edges of biological material supply-chains could help advance mycological production as a distributed manufacturing platform and contribute to a flourishing bioeconomy that benefits all people and the planet [34, 99, 100].

## Methods

### Strains

We acquired four sequenced strains of wood-degrading fungi from various sources (see Additional file 1: Table S1). Upon receipt each culture was expanded to multiple 4 °C storage plates (9 cm, VWR, Radnor, P.A.) and culture slants. Culture slants were made by pouring approximately 5–10 mL of PDYA at an angle into a 15 mL Falcon tube (VWR, Radnor, P.A.) and allowing the PDYA to solidify along the inner sidewall of the tube. Each slant is inoculated with an approximately 2 cm piece of mycelium tissue, para-filmed, and stored in darkness at room temperature.

### Strain maintenance

All strains were maintained on PDYA by transferring 5 mm plugs (Biopunch, Ted Pella, Inc. Redding, CA), from leading edge tissue to fresh PDYA plates for storage at 4 °C or into slants for storage at room temperature. When beginning an experiment, starter plates for an organism are inoculated from a common source plate or slant as indicated. All cultures for a particular experiment are initiated from a common source-plate to minimize the likelihood of spontaneous mutations impacting results.

### Pre-culture

Each organism was cultured on PDYA starter plates in darkness at 30 °C for 3 days prior to experiments. Starter plates for each experiment were initiated as indicated by either transferring a 5 mm plug from leading edge tissue of 4 °C storage plates onto fresh PDYA starter plates. Again, each experiment was initiated with a new starter plate for each organism and experimental data was not compared across experiments, mitigating concerns of variation in radial extension measurements resulting from genetic mutations that may accumulate over subsequent sub-culturing.

### Substrates

NIST RM 8492 was obtained from NIST. U.S.-based Pringles™ Original flavor were acquired from Amazon.com, assorted flavors were acquired from Walmart (Bentonville, A.R.), and Original flavor Pringles™ from the five Pringles™ global production factories were acquired from various retail stores in the countries of origin. Interlaboratory participants acquired locally-sourced Pringles™ from various local retail stores. PDYA was prepared using various laboratory grade reagents (per 0.5 L; 20 g glucose, Sigma; 10 g starch from potato, Sigma; 7.5 g Yeast Extract, EMD Millipore Corporation; 5.0 g Bacto Agar, Becton, Dickinson and Company). Yeast Synthetic Defined (YSD) media was prepared according to the manufacturer recommendations (Clontech, Takara Bio USA, Inc., Mountain View, CA). Commercially available woodchip substrate was purchased from Out Grow (IL, U.S.). Hardwood and Softwood pallets were identified as such and obtained from All Good Pallets, Inc. (Newark, C.A.) and chipped to 1–2 cm particle size in a batch process prior to preparation for experiments. Cardboard was harvested from Amazon.com boxes in the Shriram Center for Bioengineering and Chemical Engineering recycle bins.

### Substrate preparation

Agar-based aqueous extract experimental plates were made for each substrate. Each solid substrate was weighed, ground for 2.5 min in a Magic Bullet (Walmart, Bentonville, A.R.), and the ground substrate was suspended in deionized water in a glass beaker to make 2% aqueous extract solutions. Aqueous extractions were performed by boiling the substrate and deionized water mixture in an autoclave for 45 min at 121 °C. The aqueous extract was filtered under vacuum through a funnel covered by cheese cloth (Labscientific, Inc., N.J.) into an Erlenmeyer flask. Solid particulate was discarded and the liquid broth was harvested to make agar-based plates. Agar was added to the aqueous extract broth to make 1% agar solutions for all substrates (2% agar for interlaboratory studies). The aqueous extract broth and agar mixture was autoclaved at 121 °C for 30 min prior to use, allowed to cool, and 20 mL of the mixture was dispensed into each plate.

### Plate-based colony radial extension measurements

Plate-based measurements were conducted in triplicate. Experimental plates were inoculated with a 5 mm plug of leading edge tissue from starter plates and incubated in the dark, at 30 °C, in ambient humidity (Fig. 2: Compost, Manure, Woodchip, PDYA, YSD) or approximately 80% humidity (Figs. 3, 4, 5, 6, 7), until the fastest growing fungus reached the edge of the plate (approximately 4 days for *P. chrysosporium* on PDYA). While humidity levels may impact colony characteristics, such as radial growth rates, and should be controlled and considered when comparing absolute growth rate data across experiments, our use of a reference organism and relative extension units (REUs) helps mitigate effects due to differences in extrinsic factors such as humidity. Every 24 h the leading edge of each colony was traced with a marker. Measurements of distances between all colony leading edge traces were performed on the final day (Fig. 1). Measurements were obtained by hand using a ruler (Fig. 2: Compost, Manure, Woodchip, PDYA, YSD) or using image analysis software, as indicated (Figs. 3, 4, 5, 6, 7). Measurements were recorded in a CSV file and used for downstream analysis.

### Interlaboratory study measurement kit

Each participating laboratory was provided a measurement kit (see Additional file 1: Figure S2). Kits contained four fungi of interest, a coring tool for excising fungal tissue plugs, cheese cloth for filtering aqueous extracts, and centrally-provided Pringles™ substrate. The contents were packaged within the original product packaging and shipped via FedEx to participating labs within the contiguous United States. Simple instructions for conducting colony radial extension measurements were emailed to participants. Briefly, participants were instructed to culture their starter plates accordingly for 3 days prior to starting experimental plates, perform colony radius traces for 5 days, image their plates, and email the images to our group. Interlaboratory study images were processed identically to all other experimental images.

### Image analysis

Plates from experiments performed by our group were imaged using an iPhone 8. Images from our interlaboratory study were acquired by participants using their preferred method and returned to us for image processing and analysis. Images were converted to PNG using the Mac application Preview, measurement axis were superimposed (Fig. 1), and the images were subsequently analyzed in Fiji (ImageJ) [101]. The Fiji Set Scale tool was used to calibrate pixel distance to millimeters using the Petri dish diameter as a reference distance. The Measurement tool was used to measure distances between traces along predetermined superimposed axis starting from a central point on the edge of the inoculation plug (Fig. 1). Values were recorded in a spreadsheet for downstream analysis.

### Statistical analysis

All analysis was performed using custom Python scripts. The Pandas library was used for data curation [102]. Where appropriate contaminated plate data was dropped from the dataset (Fig. 5: Original-USA, two plates dropped due to contamination). Normality of data was checked using the Shapiro–Wilk test in the StatsModels module [103]. StatsModels ANOVA was used to test for significant differences within factors. Tukey HSD in StatsModels was used to identify statistically significant differences between groups. The Scikit-learn library used for feature normalization and linear regression models [104]. All plots were made using MatPlotLib and Seaborn libraries [105].

## Supplementary information

**Additional file 1.** Organisms and substrates used in this study, NIST RM 8492 reference mass fraction values, myco-metrology kit image and instruction manual, and additional REU data.

## Data Availability

Samples of *P. chrysosporium*, *T. versicolor*, and *G. lucidum* can be sourced directly from the United States Department of Agriculture, U.S. Forest Service Forest Products Lab. The JGI-sequenced strain of *S. commune* that is closely related to the strain used in this work (Han Wosten, personal communication) can be sourced directly from the Fungal Genetics Stock Center (strain ID: FGSC #9210).

## Abbreviations

NIST: National Institute of Standards and Technology
US: United States
USDA: United States Department of Agriculture
IEC: International Electrotechnical Commission
IETF: Internet Engineering Task Force
ISO: International Organization for Standardization
NCBI: National Center for Biotechnology Information
CECT: Spanish Type Culture Collection
USDA APHIS: USDA Animal and Plant Health Inspection Service
U.S. D.o.E. J.G.I.: U.S. Department of Energy Joint Genome Institute
PDYA: Potato Dextrose Yeast Extract
YSD: Yeast Synthetic Defined
P.A.: Pennsylvania
C.A.: California
A.R.: Arkansas
I.L.: Illinois
N.J.: New Jersey
UK: United Kingdom
EU: European Union

## References

[1] Langholtz, B. M. H. J., Stokes, L. M. & Eaton. 2016 Billion-Ton Report Advancing Domestic Resources for a Thriving Bioeconomy. 2016. https://www.energy.gov/sites/prod/files/2016/12/f34/2016_billion_ton_report_12.2.16_0.pdf. Accessed 22 Jan 2020

[2] Board, B. *The Bioeconomy Initiative: Implementation Framework*. 2017. https://biomassboard.gov/pdfs/Bioeconomy_Initiative_Implementation_Framework_FINAL.pdf. Accessed 22 Jan 2020.

[3] Carlson R (2016). Estimating the biotech sector’s contribution to the US economy. Nat Biotechnol.

[4] Carlson, R. Bioeconomy Capital: Bioeconomy Dashboard. 2019. https://www.bioeconomycapital.com/bioeconomy-dashboard. Accessed 22 Jan 2020.

[5] US Department of Agriculture. US Biobased Products Market Potential and Projections Through 2025, OCE-2008–01. 2008. https://www.usda.gov/oce/reports/energy/BiobasedReport2008.pdf. Accessed 22 Jan 2020.

[6] Cumbers, J. The Bio-Belt : Growing the Future in Rural America. 2019. https://www.forbes.com/sites/johncumbers/2019/07/15/the-bio-belt-growing-the-future-in-rural-america/#34369eed5461. Accessed 22 Jan 2020.

[7] Liao JC, Mi L, Pontrelli S, Luo S (2016). Fuelling the future: microbial engineering for the production of sustainable biofuels. Nat Publ Gr.

[8] Floudas D (2012). The paleozoic origin of enzymatic lignin decomposition reconstructed from 31 fungal genomes. Science.

[9] Loyd AL, Held BW, Linder ER, Smith JA, Blanchette RA (2018). Elucidating wood decomposition by four species of Ganoderma from the United States. Fungal Biol..

[10] Ohm RA (2014). Genomics of wood-degrading fungi. Fungal Genet Biol.

[11] Riley R (2014). Extensive sampling of basidiomycete genomes demonstrates inadequacy of the white-rot/brown-rot paradigm for wood decay fungi. Proc Natl Acad Sci.

[12] Grimm D, Wösten HAB (2018). Mushroom cultivation in the circular economy. Appl Microbiol Biotechnol.

[13] Higgins C, Margot H, Warnquist S, Obeysekare E, Mehta K. Mushroom cultivation in the developing world: a comparison of cultivation technologies. In: GHTC 2017 - IEEE Glob. Humanit. Technol. Conf. Proc. 2017.

[14] Bayer E. The mycelium revolution is upon us. Scientific American*.* 2019. https://blogs.scientificamerican.com/observations/the-mycelium-revolution-is-upon-us/. Accessed 22 Jan 2020.

[15] Patrick S. The human mycobiome. Cold Spring Harb. Perspect. Med*.* 2015; 103–122. 10.1101/cshperspect.a019810 PMC4448585 25384764

[16] Chang Y (2015). Phylogenomic analyses indicate that early fungi evolved digesting cell walls of algal ancestors of land plants. Genome Biol Evol.

[17] Hooker CA, Lee KZ, Solomon KV (2019). Leveraging anaerobic fungi for biotechnology. Curr Opin Biotechnol.

[18] Fredericksen MA (2017). Three-dimensional visualization and a deep-learning model reveal complex fungal parasite networks in behaviourally manipulated ants. Proc Natl Acad Sci.

[19] Pawlowska TE (2018). Biology of fungi and their bacterial endosymbionts. Annu Rev Phytopathol.

[20] Marquez LM, Redman RS, Rodriguez RJ, Roossinck MJ (2007). A virus in a fungus in a plant: three-way symbiosis required for thermal tolerance. Science.

[21] Peay KG, Kennedy PG, Talbot JM (2016). Dimensions of biodiversity in the Earth mycobiome. Nat Rev Microbiol.

[22] Riquelme M (2011). Architecture and development of the *Neurospora crassa* hypha—a model cell for polarized growth. Fungal Biol.

[23] Moore D, Robson G, Trinci T (2011). 21st century guidebook to fungi.

[24] Roper M, Lee CH, Hickey PC, Gladfelter AS (2015). Life as a moving fluid: Fate of cytoplasmic macromolecules in dynamic fungal syncytia. Curr Opin Microbiol.

[25] Kang X (2018). Molecular architecture of fungal cell walls revealed by solid-state NMR. Nat Commun.

[26] Kiss E (2019). Comparative genomics reveals the origin of fungal hyphae and multicellularity. Nat Commun..

[27] Roper M, Dressaire E (2019). Fungal biology: bidirectional communication across fungal networks. Curr Biol.

[28] Fleißner A, Herzog S (2016). Signal exchange and integration during self-fusion in filamentous fungi. Semin Cell Dev Biol.

[29] Lehmann, A., Zheng, W., Soutschek, K. & Rillig, M. C. How to build a mycelium: tradeoffs in fungal architectural traits. *bioRxiv.* 2018; 361253.

[30] Harris SD (2019). Hyphal branching in filamentous fungi. Dev Biol.

[31] Gehrmann T (2018). Nucleus-specific expression in the multinuclear mushroom-forming fungus *Agaricus bisporus* reveals different nuclear regulatory programs. Proc Natl Acad Sci.

[32] Krizsán K (2019). Transcriptomic atlas of mushroom development reveals conserved genes behind complex multicellularity in fungi. Proc Natl Acad Sci.

[33] Varga T (2019). Megaphylogeny resolves global patterns of mushroom evolution. Nat Ecol Evol.

[34] Tsing AL (2015). The mushroom at the end of the world: on the possibility of life in capitalist ruins. The mushroom at the end of the world.

[35] Kauffman, J. There ’s a new source for meat substitutes : fungi. *WSJ*. 2019. https://www.wsj.com/articles/theres-a-new-source-for-meat-substitutes-fungi-11570647109. Accessed 22 Jan 2020.

[36] Li X (2019). Transcriptional profiling of *Auricularia cornea* in selenium accumulation. Sci Rep.

[37] Mir-Tutusaus JA (2019). Long-term continuous treatment of non-sterile real hospital wastewater by *Trametes versicolor*. J Biol Eng.

[38] Xia M-C (2018). Isolation and identification of *Penicillium chrysogenum* strain Y5 and its copper extraction characterization from waste printed circuit boards. J Biosci Bioeng.

[39] Ecovative Design LLC. We grow materials. 2018. https://ecovativedesign.com/. Accessed 22 Jan 2020.

[40] MycoWorks. We turn mycelium and agricultural byproducts into leather. https://www.mycoworks.com. Accessed 22 Jan 2020.

[41] Jones M (2018). Thermal degradation and fire properties of fungal mycelium and mycelium—biomass composite materials. Sci Rep.

[42] Mazur, R. Mechanical Properties of Sheets Comprised of Mycelium : A Paper Engineering Perspective. *Thesis* (2015).

[43] Hao J (2018). Bio-templated fabrication of three-dimensional network activated carbons derived from mycelium pellets for supercapacitor applications. Sci Rep.

[44] Campbell B, Ionescu R, Favors Z, Ozkan CS, Ozkan M (2015). Bio-derived, binderless, hierarchically porous carbon anodes for li-ion batteries. Sci Rep..

[45] Stamets PE (2018). Extracts of polypore mushroom mycelia reduce viruses in honey bees. Sci Rep.

[46] Molnár Z (2018). Green synthesis of gold nanoparticles by thermophilic filamentous fungi. Sci Rep.

[47] Jones M, Huynh T, Dekiwadia C, Daver F, John S (2017). Mycelium composites: a review of engineering characteristics and growth kinetics. J Bionanoscience.

[48] Jones M, Huynh T, John S (2018). Inherent species characteristic influence and growth performance assessment for mycelium composite applications. Adv Mater Lett.

[49] Islam MR, Tudryn G, Bucinell R, Schadler L, Picu RC (2017). Morphology and mechanics of fungal mycelium. Sci Rep..

[50] Appels FVW (2018). Hydrophobin gene deletion and environmental growth conditions impact mechanical properties of mycelium by affecting the density of the material. Sci Rep.

[51] Appels FVW (2019). Fabrication factors influencing mechanical, moisture- and water-related properties of mycelium-based composites. Mater Des.

[52] Haneef M (2017). Advanced materials from fungal mycelium: fabrication and tuning of physical properties. Sci Rep.

[53] Sun W, Tajvidi M, Hunt CG, McIntyre G, Gardner DJ (2019). Fully bio-based hybrid composites made of wood, fungal mycelium and cellulose nanofibrils. Sci Rep.

[54] Chang J (2019). Modified recipe to inhibit fruiting body formation for living fungal biomaterial manufacture. PLoS ONE.

[55] Cerimi K, Akkaya KC, Pohl C, Schmidt B, Neubauer P (2019). Fungi as source for new bio-based materials: a patent review. Fungal Biol Biotechnol.

[56] Goffeau A (1996). Life with 6000 genes. Science.

[57] Staben C (2003). The genome sequence of the filamentous fungus *Neurospora crassa*. Nature.

[58] Pel HJ (2007). Genome sequencing and analysis of the versatile cell factory *Aspergillus niger* CBS 513.88. Nat Biotechnol..

[59] Chen S (2012). Genome sequence of the model medicinal mushroom *Ganoderma lucidum*. Nat Commun.

[60] Binder M (2013). Phylogenetic and phylogenomic overview of the Polyporales. Mycologia.

[61] Stajich JE (2010). Insights into evolution of multicellular fungi from the assembled chromosomes of the mushroom *Coprinopsis cinerea* (*Coprinus cinereus*). Proc Natl Acad Sci.

[62] Morin E (2013). Genome sequence of the button mushroom Agaricus bisporus reveals mechanisms governing adaptation to a humic-rich ecological niche. Proc Natl Acad Sci USA.

[63] Ohm RA (2010). Genome sequence of the model mushroom Schizophyllum commune. Nat. Biotechnol..

[64] Martinez D (2004). Genome sequence of the lignocellulose degrading fungus *Phanerochaete chrysosporium* strain RP78. Nat Biotechnol.

[65] Huang Y-H (2013). Generation and analysis of the expressed sequence tags from the mycelium of *Ganoderma lucidum*. PLoS ONE.

[66] Yu GJ (2012). Deep insight into the *Ganoderma lucidum* by comprehensive analysis of its transcriptome. PLoS ONE.

[67] Ma Z (2018). Reconstruction and analysis of a genome-scale metabolic model of Ganoderma lucidum for improved extracellular polysaccharide production. Front Microbiol.

[68] Elsacker E, Vandelook S, Brancart J, Peeters E, De Laet L (2019). Mechanical, physical and chemical characterisation of mycelium-based composites with different types of lignocellulosic substrates. PLoS ONE.

[69] Surowiecki, J. Turn of the century. *WIRED.* (2002) https://www.wired.com/2002/01/standards-2/. Accessed 22 Jan 2020.

[70] Duncan, T. Before the Melting Pot : Pre- Columbian Weights and Measures. *NIST.gov* (2019). https://www.nist.gov/blogs/taking-measure/melting-pot-pre-columbian-weights-and-measures. Accessed 22 Jan 2020.

[71] Vincent, N. The clauses of Magna Carta. *British Library* 2015. https://www.bl.uk/magna-carta/articles/the-clauses-of-magna-carta. Accessed 22 Jan 2020.

[72] U.S Constitution. 1787. https://guides.loc.gov/constitution. Accessed 22 Jan 2020.

[73] Arkin AP, Endy D. A standard parts list for biological circuitry. 1999. https://dspace.mit.edu/handle/1721.1/29794. Accessed 22 Jan 2020.

[74] Knight T. Idempotent vector design for standard assembly of biobricks. 2003. https://dspace.mit.edu/handle/1721.1/21168. Accessed 22 Jan 2020.

[75] Endy D, Brent R (2001). Modelling cellular behaviour. Nature.

[76] Endy D (2005). Foundations for engineering biology. Nature.

[77] Canton B, Labno A, Endy D (2008). Refinement and standardization of synthetic biological parts and devices. Nat Biotechnol.

[78] Shetty RP, Endy D, Knight TF (2008). Engineering BioBrick vectors from BioBrick parts. J Biol Eng.

[79] Kelly JR (2009). Measuring the activity of BioBrick promoters using an in vivo reference standard. J Biol Eng.

[80] Galdzicki M (2014). The Synthetic Biology Open Language (SBOL) provides a community standard for communicating designs in synthetic biology. Nat Biotechnol.

[81] Brandl J (2018). A community-driven reconstruction of the *Aspergillus niger* metabolic network. Fungal Biol Biotechnol.

[82] Idnurm A, Meyer V (2018). The CRISPR revolution in fungal biology and biotechnology, and beyond. Fungal Biol Biotechnol.

[83] Cairns TC, Zheng X, Zheng P, Sun J, Meyer V (2019). Moulding the mould: understanding and reprogramming filamentous fungal growth and morphogenesis for next generation cell factories. Biotechnol Biofuels.

[84] Cairns TC, Nai C, Meyer V (2018). How a fungus shapes biotechnology: 100 years of *Aspergillus niger* research. Fungal Biol Biotechnol..

[85] Baker T (2001). Leaf litter decomposition and nutrient dynamics in four southern forested floodplain communities. Soil Sci Soc Am J.

[86] Meier CL, Rapp J, Bowers RM, Silman M, Fierer N (2010). Fungal growth on a common wood substrate across a tropical elevation gradient: temperature sensitivity, community composition, and potential for above-ground decomposition. Soil Biol Biochem.

[87] ASTM D1413-07e1, Standard test method for wood preservatives by laboratory soil-block cultures. *ASTM Int.* 2007.

[88] Keuskamp JA, Dingemans BJJ, Lehtinen T, Sarneel JM, Hefting MM (2013). Tea Bag Index: a novel approach to collect uniform decomposition data across ecosystems. Methods Ecol Evol.

[89] Macdonald E (2018). Using the Tea Bag Index to characterize decomposition rates in restored peatlands.

[90] 2^nd^ International Tea Bag Index Workshop. *European Soil Data Centre (ESDAC)*. https://esdac.jrc.ec.europa.eu/event/2nd-international-tea-bag-index-workshop. Accessed 22 Jan 2020.

[91] teatime4science. Welcome to the Teabag Index website! 2016. https://www.teatime4science.org. Accessed 22 Jan 2020.

[92] Klein J. A mycologist hopes to show how a simple, silly experiment can illuminate fungal biology. 2019. https://www.nytimes.com/2019/03/29/science/marshmallow-peeps-fungus.html. Accessed 22 Jan 2020.

[93] Wise SA, Watters Jr. RL. *NIST RM 8492 Report of Investigation*. 2011.

[94] Templeton DW, Wolfrum EJ, Yen JH, Sharpless KE (2016). Compositional analysis of biomass reference materials: results from an interlaboratory study. Bioenergy Res.

[95] Pringles. 2019. https://www.potatopro.com/brands/pringles. Accessed 22 Jan 2020.

[96] Naidu, R. Kellogg, Mondelez stock up on Pringles, Milka for fear of Brexit. *Reuters.* 2019. https://www.reuters.com/article/us-britain-eu-kellogg/kellogg-mondelez-stock-up-on-pringles-milka-for-fear-of-brexit-idUSKCN1Q81XB. Accessed 22 Jan 2020.

[97] De Ligne L (2019). Analysis of spatio-temporal fungal growth dynamics under different environmental conditions. IMA Fungus.

[98] Calles J, Justice I, Brinkley D, Garcia A, Endy D (2019). Fail-safe genetic codes designed to intrinsically contain engineered organisms. Nucleic Acids Res.

[99] Haraway D (2016). Staying with the trouble: making kin in the Chthulucene.

[100] Haraway D, Endy D, Lejeune L (2019). Tools for multispecies futures. J Des Sci.

[101] Schindelin J (2012). Fiji: an open-source platform for biological-image analysis. Nat Methods.

[102] Mckinney W. Data structures for statistical computing in python. In: Proc. 9th Python Sci. Conf. (SCIPY 2010). 2010.

[103] Seabold S, Perktold J, Statsmodels: econometric and statistical modeling with Python. In: 9th Python Sci. Conf. 2010. p. 57–61.

[104] Pedrogosa F, Varoquaux G, Gramfort A, Michel V (2011). Scikit-learn: machine learning in python. J Mach Learn Res.

[105] Hunter JD (2007). Matplotlib: A 2D graphics environment. Comput Sci Eng.

