# Supplementary material for: Enabling community-based metrology for wood-degrading fungi
Source: Fungal Biol Biotechnol. 2020 Mar 19;7:2. doi: 10.1186/s40694-020-00092-2 (PMC7081594; doi:10.1186/s40694-020-00092-2)
Supplement: Supplementary file 1 — Additional file 1. Organisms and substrates used in this study, NIST RM 8492 reference mass fraction values, myco-metrology kit image and instruction manual, and additional REU data. [file 40694_2020_92_MOESM1_ESM.pdf]

Enabling community-based metrology for wood-degrading fungi

Rolando Perez[1], Marina Luccioni[1], Rohinton Kamakaka[2], Samuel Clamons[3,4], Nathaniel Gaut[5], Finn Stirling[6,7], Katarzyna P. Adamala[5], Pamela A. Silver[6,7], Drew Endy[1*]

Supplementary Information Table of Contents

FIGURE S1. PLATE-BASED ASSAYS ENABLE CHARACTERIZATION OF VARIOUS PARAMETERS OF FILAMENTOUS FUNGI PHYSIOLOGY. 2

TABLE S1. ORGANISMS USED IN THIS STUDY. 3

TABLE S2. SUBSTRATES USED IN THIS STUDY. 4

TABLE S3. REFERENCE MASS FRACTION VALUES FOR CONSTITUENTS IN RM 8492. 5

FIGURE S2. MYCO-METROLOGY KIT V1. 6

MYCO-METROLOGY KIT V1 INSTRUCTION MANUAL 7

FIGURE S3. COMMUNITY-BASED MEASUREMENTS OF RADIAL EXTENSION ON MEDIA MADE FROM LOCALLY-SOURCED AND CENTRALLY-PROVIDED PRINGLES™ ARE WITHIN 1.5-FOLD 10

FIGURE S4. REPORTING RELATIVE EXTENSION UNITS OF ORGANISMS GROWN ON CENTRALLY-PROVIDED PRINGLES™ REDUCES VARIATION BETWEEN MEASUREMENTS ACROSS LOCATIONS. 11

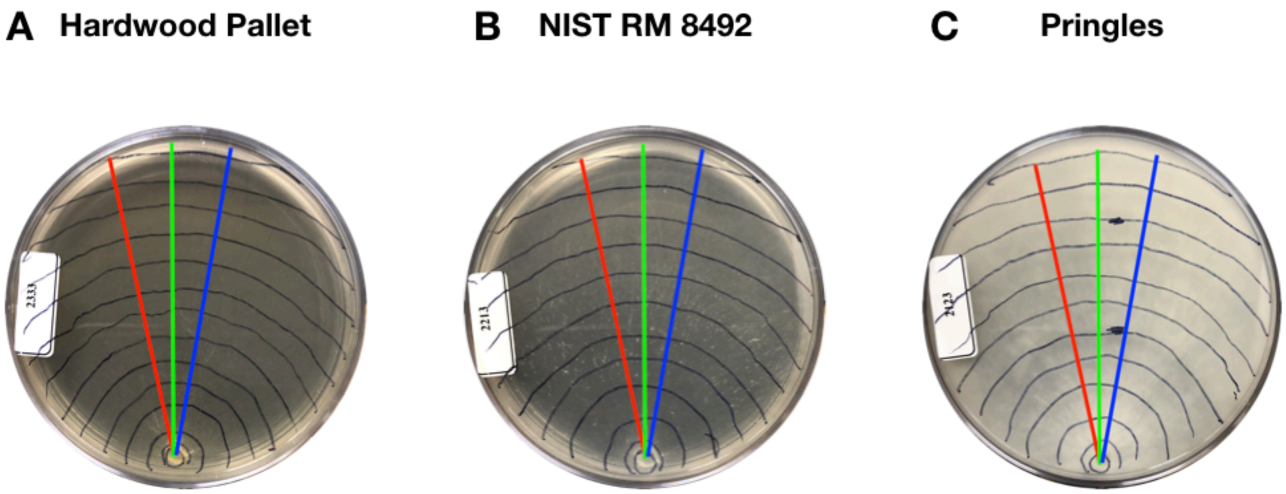

Figure S1. Plate-based assays enable characterization of various parameters of filamentous fungi physiology. In addition to radial extension, density of aerial hyphae, pigmentation, branch rate, and other important characteristics of filamentous fungi can be qualitatively and qualitatively determined using plate-based assays. We observed qualitative differences in mycelium density and aerial hyphae density depending on the substrate composition. Pictured here are representative images of *G. lucidum*.

Name and Strain ID	Image	Source
Phanerochaete chrysosporium **(RP-78)**	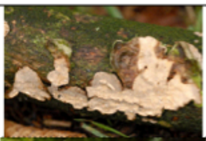	**Forest Products Lab**
Schizophyllum commune **(4.8B)**	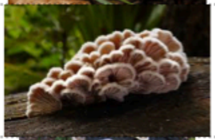	**Han Wosten**
Trametes versicolor **(Fp-101664-Spp)**	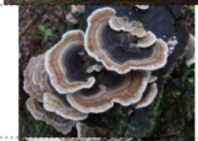	**Forest Products Lab**
Ganoderma lucidum **(10597-SSI)**	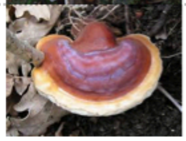	**Forest Products Lab**

Table S1. Organisms used in this study. The left column indicates the name and strain ID of each organism, the center column contains images of the organisms as they are found in nature, and the right column indicates the source of the strain. The strain ID for *P. chrysosporium*, *T. versicolor*, and *G. lucidum* can be used to explore the genome assemblies and annotations via the U.S. Department of Energy's Joint Genome Institute fungal genomics resource, MycoCosm. *S. commune* strain (4.8B) used in this study is closely-related to the JGI-sequenced strain (H4-8) whose genome assembly and annotation is also available via MycoCosm (https://mycocosm.jgi.doe.gov/mycocosm/home).

Name	Image	Source
Compost	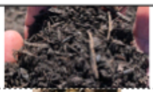	Stanford Farm; Regen, Inc.
Manure	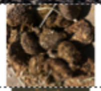	Stanford Barn
Wood Chip	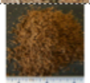	Out Grow, Inc.
PDYA	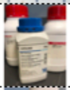	Sigma Aldrich
YSD	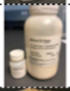	Clontech; Takara Bio USA, Inc.
NIST RM 8492	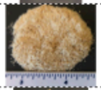	NIST
Softwood Pallet	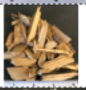	All Good Pallets, Inc.
Hardwood Pallet	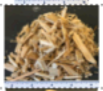	All Good Pallets, Inc.
Cardboard	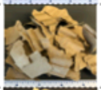	Amazon, Inc.
Pringles	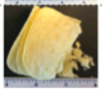	Kellogg, Inc.

Table S2. Substrates used in this study. We sourced various substrates that are either commonly-used, laboratory-grade, fully-traceable, or widely-available. The left column indicates the substrate name, the center column shows representative images of the substrates, and the right column indicates the source of the substrate.

Table 1. Reference Mass Fraction Values for Constituents in RM 8492

	Mass Fraction (%)	Expanded Uncertainty (%)	Coverage Factor, *k*
Water Extractives	2.9	1.2	2.20
95 % Ethanol Extractives (after water extraction)	1.54	0.63	2.20
Sucrose	0.045	0.046	2.20
Whole Ash	0.96	0.23	2.18
Extractives-Free Ash	0.741	0.077	2.26
Glucan	44.6	1.2	2.18
Xylan	13.39	0.39	2.18
Arabinan	0.35	0.30	2.18
Galactan	0.55	0.53	2.20
Mannan	2.16	0.30	2.18
Structural Sugars	61.0	1.8	2.18
Total Lignin	27.2	1.3	2.18
Acid-Insoluble Residue	24.0	1.2	2.20
Acid-Soluble Lignin	2.2	1.7	2.20
Acetyl	3.3	1.8	2.23
Nitrogen	0.17	0.11	2.31
Total Component Closure	99.4	1.5	2.26

Table S3. Reference Mass Fraction Values for Constituents in RM 8492. The entire table and all values shown here are reproduced directly from the NIST Report of Investigation for Reference Material 8492 Eastern Cottonwood Whole Biomass Feedstock[92]. NIST RM 8492 Eastern Cottonwood Whole Biomass Feedstock is fully-traceable and compositionally-characterized candidate reference material. RM 8492 is intended primarily for use in evaluating analytical methods for the determination of summative composition of lignocellulosic material. The RM can also be used for quality assurance when assigning values to in-house control materials.

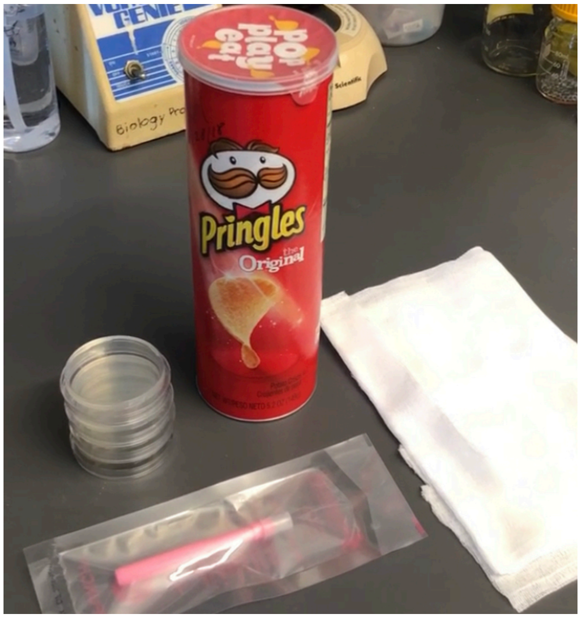

Figure S2. Myco-Metrology Kit v1. We made kits (pictured) containing centrally-provided Pringles™, fungal cultures, a coring tool, and cheese cloth.

Myco-Metrology Kit v1 Instruction Manual

Hello Intrepid Explorer,

You are the lucky recipient of an experimental MycoKit.

Have a quick look at this video for an overview of a similar process for deploying your MycoKit in support of reliable mycelium-based distributed bioproduction.

- watch

Tread **heavily** and press onward into the unknown!

/Rolando

————protocol————

Kit contents:

- Pringles
- strains (6x)
- cheese cloth
- tissue coring tool

Materials needed but not included:

- 1L glass beaker (2x)
- 1L glass bottles (2x)
- 250 mL glass beaker
- 1 L graduated cylinder
- vacuum filter flask
- funnel
- parafilm
- 9 cm petri dishes (36x)
- agar
- diH2O
- 70% ethanol spray bottle
- sticker labels
- Sharpie marker
- large sponge
- magic bullet, blender, or other grinding tool
- camera or camera phone
- analytical balance
- microwave
- incubator
- autoclave
- lab bench vacuum supply line
- bunsen burner and lab bench gas supply line

Overview: Please use the contents of your MycoKit and a can of Pringles Original flavor that you have purchased from your local grocery store to measure growth rate of each strain on the two different Pringles substrates.

Procedure (3-4 hours for setup, 1 hour per day for 7 days, 1 hour imaging on final day, total hours: 12 hours):

1. Unpack your kit and take inventory.
2. Take inventory of the “required but not included” materials and equipment.
3. **Prepare the substrate for aqueous extraction (steps 3 & 4, ~20 minutes):** Weigh 14 grams of the substrate and grind the substrate for 2.5 minutes in the magic bullet, or using a blender or mortar and pestle until the substrate is pulverized.
4. **Setup aqueous extraction (~5 minutes):** In a 1L glass beaker add pulverized substrate and 700 mL of diH2O and autoclave at 121C for 45 minutes using standard safety precautions and standard autoclave tape
5. **Build your filtration system (steps 5-8, 15 minutes):** While the aqueous extraction process it taking place in the autoclave, prepare the filtration system by first cutting the provided cheese cloth into 4 equal sized pieces.
6. Set up your vacuum filter funnel by lining the outer surface of the funnel neck with parafilm.
7. Using one piece of the cheese cloth, fold the cloth such that it will fit into the funnel without being pushed all the way through the funnel neck. Position the funnel into the flask, forming a tight seal.
8. Connect the vacuum filter neck of the flask to the lab bench vacuum line.
9. **Collect aqueous extraction (~30 mins total):** Pour the autoclaved substrate into the funnel, allowing time for the liquid aqueous extract portion to flow through the cheese cloth in the funnel and into the flask. Dispose of the solid materials that are captured by the cheese cloth in the funnel.
    - **Note:** If the cheese cloth becomes clogged, replace the cheese cloth with a new piece.
10. **Prepare substrate mix (steps 10-11, ~15 minutes):** Add 20 grams of agar to a 1 L glass bottle.
11. Using the 1 L graduated cylinder, measure 500 mL of the aqueous extract and pour into the 1 L glass bottle containing the 20 grams of agar. Autoclave the bottle with aqueous extract and agar mix at 121C for 30 minutes using standard safety precautions and standard autoclave tape.
12. **Pour plates (~1 hour):** Under bench-top sterile conditions, using your bunsen burner, pour 20 mL/plate of the aqueous extract + agar mixture into ~25 Petri dish plates. Allow the plates to solidify under sterile conditions.
13. Repeat steps 3-12 for each substrate.
14. **Plug your plates (~45 minutes total):** Under sterile conditions, using sterile technique to maintain sterility of your tissue coring tool when working with each plug and taking care to prevent cross contamination of strains, you will inoculate 3 plates for each substrate for each strain in the following manner:
    - **Note:** To sterilize your tissue coring tool and forceps simply spray the tips with 70% ethanol solution and flame very briefly. Take care not to melt your coring tool. If you do melt it a little, no biggie just press on with your tool trying not to melt it completely.
    - Using your 5 mm tissue coring tool cut 6 plugs of tissue into the leading edge of the growing starter colony, such that 75% of the tissue plug surface is covered in mycelium tissue.
    - Using your forceps, pick one tissue plug and place the plug, mycelium tissue facing down, onto the agar surface at one edge of the plate — keeping the agar surface facing upright.
    - Mark the location of the tissue plug onto the bottom surface of the plate.
    - Repeat 6 times for each strain: 3 plates per substrate/per strain, for 6 strains, 18 plates per substrate, for a total of 36 plates.
    - Sterilize your tissue coring tool and forceps in between work with each strain.

15. Incubate all plates, agar surface facing upright, at 30°C and ~80% humidity. To control humidity, simply fill 250 mL beaker with 50 mL of water and soak the large sponge with the water. Place the beaker with the residual water and the damp sponge inside into the incubator, as close as possible to the 36 plates.
16. **Colony edge tracing (~1 hour):** Every 24 hours use your Sharpie to trace the leading edge profile of each growing colony. Repeat for 7 days.
    - **Note:** To help visualize the colony edge against the substrate hold the plate up the ceiling lights and trace the edge profile onto the bottom surface of the plate.
17. **Image your plates (~30 minutes):** Image the bottom surface of each plate using your camera such that each image contains the full profile of the plate and is taken equidistant from its plate. We recommend you setup a camera support, perhaps an old 1000 uL pipette tip box if using a camera phone, so you can make sure each image is taken at an equal distance from its plate.
18. Send the images to rcperez@stanford.edu.
19. We crunch the numbers and share the results!

Strains

6 sequenced strains of wood-degrading fungi spanning the order Agaricales from various domestic and international sources. Please see table 1 for strain ID and source.

**Name**	**ID**	**Origin**	**Hyphae Type**
Ganoderma lucidum	10597-SSI	JGI, Forest Products Lab	1
Phanerochaete chrysosporium	RP-78	JGI, Forest Products Lab	2
Schizophyllum commune A	4.8A	Hans Wosten, UTrecht	1
Schizophyllum commune B	4.8B	Hans Wosten, UTrecht	1
Trametes versicolor SSI	FP101664-SSI	JGI, Forest Products Lab	1
Trametes versicolor Spp	FP101664-Spp	JGI, Forest Products Lab	1

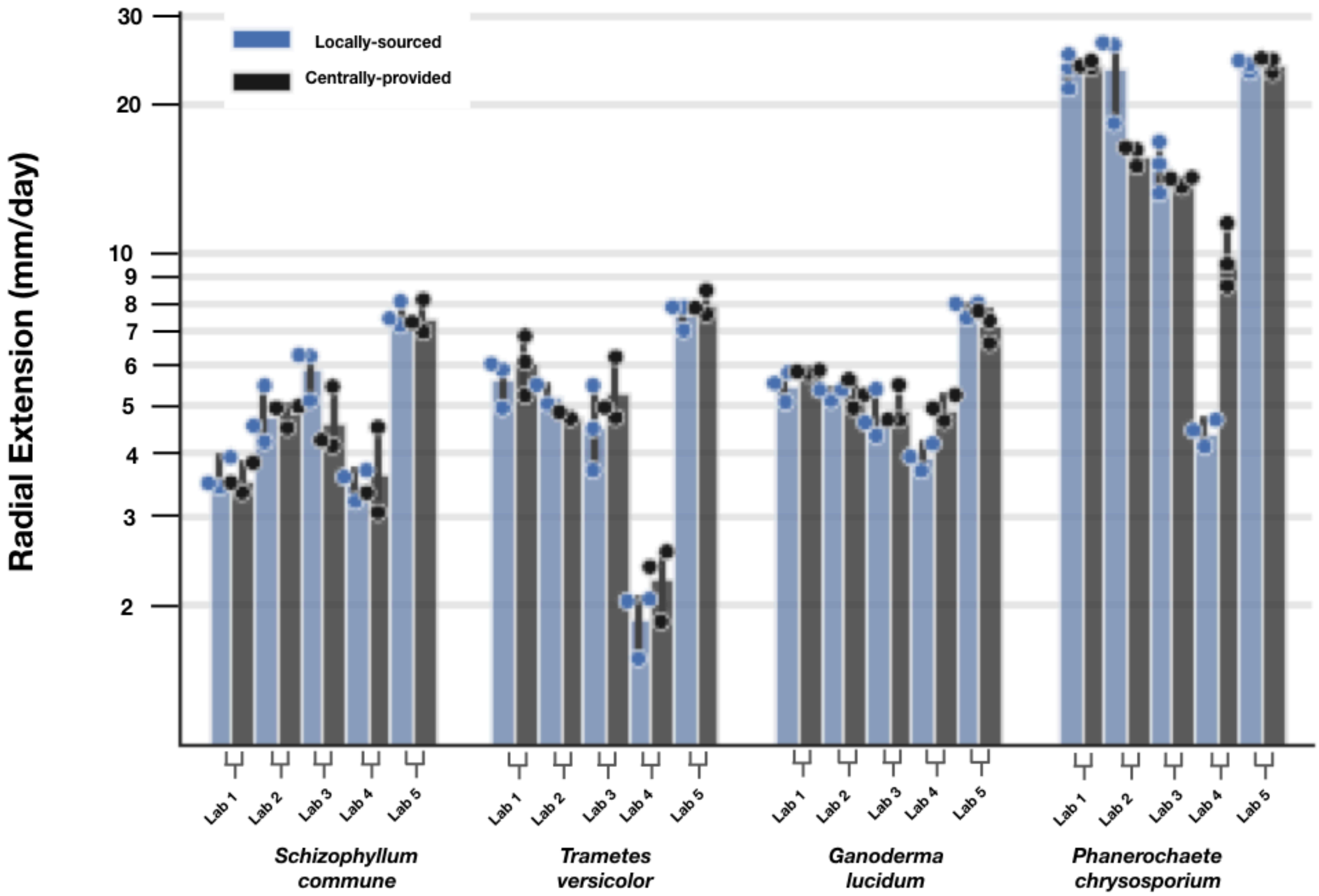

Figure S3. Community-based measurements of radial extension on media made from locally-sourced and centrally-provided Pringles™ are within 1.5-fold. Radial extension rates for individual plates (points, n=3), mean extension across all plates (bar height), and standard deviation across all plates (error bar) are shown. Statistically significant differences per Tukey's HSD ($p<0.05$) were detected for Lab 4 measurements of *P. chrysosporium* extension rates across locally-sourced and centrally-provided substrates. Statistically significant differences per Tukey's HSD ($p<0.05$) were detected across labs when using centrally-provided Pringles™ between Lab 4 and Lab 5, Lab 4 and Lab 2, Lab 5 and Lab 3, and Lab 5 and Lab 2. Statistically significant differences per Tukey's HSD ($p<0.05$) were detected across labs when using locally-sourced Pringles™ between Lab 4 and all labs (Lab 1, Lab 2, Lab 3, Lab 5), Lab 5 and Lab 3, and Lab 3 and Lab 2.

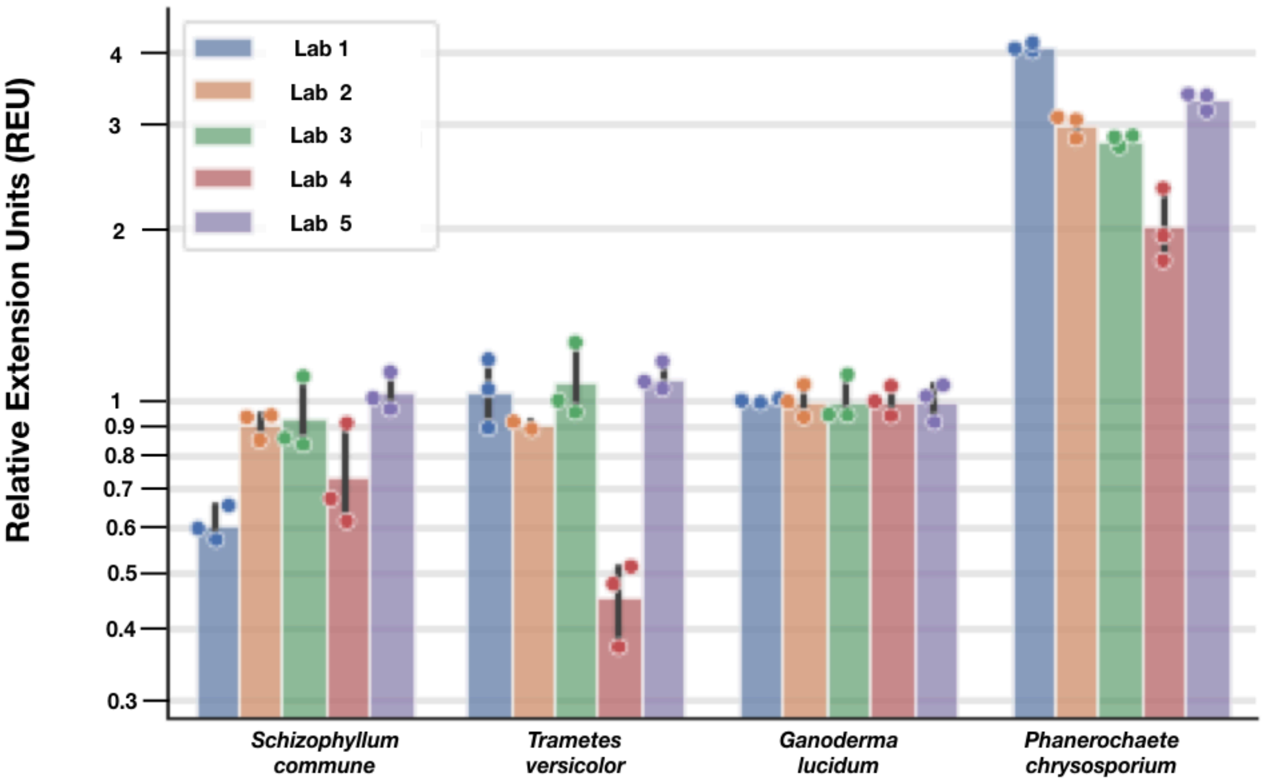

Figure S4. Reporting relative extension units of organisms grown on centrally-provided Pringles™ reduces variation between measurements across locations. Using the data from Figure 6 of organisms grown on centrally-provided Pringles™, we divided each lab's reported extension rates for a particular organism by the lab's reported extension rate for *G. lucidum*. Reported Relative Extension Units (REU) for each organism are within 2-fold with the exception of REU reported by Lab 4 for *T. versicolor.* Reporting REUs reduced coefficients of variation for *G. lucidum* by 63%, for *S. commune* by 33%, for *T. versicolor* by 22%, and for *P. chrysosporium* by 32%. Radial extension rates for individual plates (points, n=3; n=2 for Lab 2 measurements of *T. versicolor* due to contamination), mean extension rates across all plates (bar height), and standard deviation across all plates (error bar) are shown.
